# Trends and factors associated with complementary feeding practices in Ethiopia from 2005 to 2016

**DOI:** 10.1111/mcn.12926

**Published:** 2019-12-12

**Authors:** Kedir Y. Ahmed, Andrew Page, Amit Arora, Felix Akpojene Ogbo

**Affiliations:** ^1^ Translational Health Research Institute Western Sydney University, Campbelltown Campus Penrith New South Wales Australia; ^2^ College of Medicine and Health Sciences Samara University Samara Ethiopia; ^3^ School of Science and Health Western Sydney University, Campbelltown Campus Penrith New South Wales Australia; ^4^ Oral Health Services, Sydney Local Health District and Sydney Dental Hospital NSW Health Surry Hills New South Wales Australia; ^5^ Discipline of Child and Adolescent Health, Sydney Medical School, Faculty of Medicine and Health The University of Sydney Sydney New South Wales Australia; ^6^ General Practice Unit Prescot Specialist Medical Centre Makurdi Nigeria

**Keywords:** complementary feeding, infants and young children, minimum acceptable diet, minimum dietary diversity, minimum meal frequency; Ethiopia

## Abstract

Introducing appropriate complementary feeding at 6 months of age is crucial for the optimal growth and development of an infant. In Ethiopia, however, no previous national‐level studies have examined the trends and associated factors of complementary feeding practices. The aim of this study is to investigate the trends and determinants of complementary feeding practices in Ethiopia from 2005 to 2016. The study was conducted using the Ethiopia Demographic and Health Survey (EDHS) data for 2005 (*N* = 2,520), 2011 (*N* = 2,850), and 2016 (*N* = 2,864). Percentage point changes in complementary feeding indicators were estimated to examine the trends over the EDHS years. Multivariate logistic regression was used to examine the association between socioeconomic, demographic, health service, and community‐level factors and (a) the introduction of complementary foods, (b) minimum dietary diversity (MDD), (c) minimum meal frequency (MMF), and (d) minimum acceptable diet (MAD). The proportion of mothers who met MDD increased from 6.3% to 13.5% (*p* < .001), and MAD increased from 4.1% to 7.1% (*p* = .003) from 2005 to 2016. Improvements in the introduction of complementary foods (from 50.3% to 59.5%, *p* = .051) and MMF (from 41.3% to 43.6%, *p* = .288) were not statistically significant. Maternal education and occupation were associated with the introduction of complementary foods, MDD, MMF, and MAD. Higher partner education and frequent antenatal visits were associated with MDD and MAD. Children whose mothers listened to the radio had higher odds of MDD, MMF, and MAD. Our analysis of the EDHS suggests that the proportion of MDD and MAD were unacceptably low. Interventions aiming to improve complementary feeding practices in Ethiopia should also target mothers with low education, antenatal service usage, and media exposure.

**Key messages**

- The study showed that there were improvements in the proportion of children who met minimum dietary diversity (MDD) and minimum acceptable diet (MAD) in Ethiopia between 2005 and 2016.
- Higher maternal and paternal education, mother's informal occupation, frequent antenatal care (ANC) visits, and listening to the radio were associated with MAD among children aged 6–23 months.
- Efforts to improve complementary feeding practices should focus on mothers with lower education and employment, infrequent ANC visits, and less exposure to the media in Ethiopia.

**Abbreviations**
ANC: antenatal care
CI: confidence interval
CSA: Central Statistics Agency
DHS: Demographic and Health Survey
EA: enumeration areas
EDHS: Ethiopian Demographic and Health Survey
ICF: Inner City Fund
IYCF: infant and young child feeding
MAD: minimum acceptable diet
MDD: minimum dietary diversity
MMF: minimum meal frequency
SDG: Sustainable Development Goals
SNNPR: Southern Nations Nationalities and Peoples Region
WHO: World Health Organization.

## 1 INTRODUCTION

The introduction of appropriate complementary feeding around the age of 6 months following exclusive breastfeeding plays a crucial role in optimal growth and development of an infant (Black et al., 2008; Heidkamp, Ayoya, Teta, Stoltzfus, & Marhone, 2015). This is because the breast milk is no longer sufficient to meet the nutritional and developmental requirements of the infant (Arikpo, Edet, Chibuzor, Odey, & Caldwell, 2018; Black et al., 2008). In contrast, inappropriate complementary feeding (such as introducing complementary foods too early or too late, or providing a less diverse diet and/or infrequent feeding) in the early years can result in short‐ and long‐term adverse health outcomes (Abeshu, Lelisa, & Geleta, 2016; WHO, 2009). The short‐term adverse health outcomes may include stunted growth (Lassi, Das, Zahid, Imdad, & Bhutta, 2013), increased risk of diarrhoeal diseases (Ogbo et al., 2017; Ogbo, Page, Idoko, Claudio, & Agho, 2016), micronutrient deficiencies, and increased risk of mortality (Ahmed, Prendiville, & Narayan, 2016; Black et al., 2013). In the long‐term, inappropriate complementary feeding can negatively affect cognitive development (Dewey & Adu‐Afarwuah, 2008; Woldehanna, Behrman, & Araya, 2017), work capacity (Martins et al., 2011), childbirth outcomes (Victora et al., 2008), and social–emotional interactions (Metwally et al., 2016)

The World Health Organization (WHO) and the United Nations Children's Fund (UNICEF) recommend the introduction of adequate, safe, and appropriate complementary foods for infants aged 6–8 months to meet nutritional and developmental needs (WHO, 2009; WHO & UNICEF, 2003). However, reports from low‐ and middle‐income countries (LMICs) indicated that many infants and young children are not receiving appropriate complementary foods (Aguayo, 2017; Black et al., 2013). For example, a study conducted in 46 LMICs showed that the proportion of infants and young children who met the minimum acceptable diet (MAD) were 16% in Africa and 26% in Asia (Lutter et al., 2011). In Africa, previous studies from Nigeria (Ogbo, Page, Idoko, Claudio, & Agho, 2015), Tanzania (Ogbo, Ogeleka, & Awosemo, 2018; Victor, Baines, Agho, & Dibley, 2014), and Francophone and Anglophone Western African countries (Issaka et al., 2015a; Issaka et al., 2015b) have reported that complementary feeding practices were below expected levels.

In Ethiopia, inadequate complementary feeding practices are a major contributor to childhood malnutrition such as stunting (Abeway, Gebremichael, Murugan, Assefa, & Adinew, 2018; Tessema, Belachew, & Ersino, 2013). Despite the decreasing trends in stunting in the last two decades in Ethiopia (from 67% in 2000 to 38% in 2016), more than five million children remain stunted (Wirth et al., 2017). Studies conducted in Ethiopia indicated that homemade complementary foods were inadequate in calories and micronutrients, with limited animal protein and poor intake of fruits and vegetables (Abeshu et al., 2016; Asres, Nana, & Nega, 2018). This inappropriate feeding practice potentially has huge short‐ and long‐term implications for child growth and development in Ethiopia. A recent systematic review focused on the introduction of complementary foods suggested that maternal employment, improved knowledge on complementary feeding, frequent antenatal care (ANC) or postnatal care visits, and giving birth at the health facility were associated with the timely introduction of complementary foods (Habtewold et al., 2018).

No previous nationally representative studies from Ethiopia have investigated trends in WHO/UNICEF complementary feeding indicators, nor has there been a comprehensive assessment of determining factors that make Ethiopian mothers introduce complementary foods too early or too late and provide a less diverse food to and/or infrequently feed their infants and young children. Detailed information on trends and determinants of timely introduction of diverse, frequent, and acceptable diet complementary foods to infants and young children would be helpful to researchers, health practitioners, and policymakers and can inform policy interventions. Furthermore, context‐specific evidence from Ethiopia will be essential to international stakeholders given the promotion of appropriate complementary feeding in LMICs such as Ethiopia in the United Nations Decade of Action on Nutrition (2016–2025, reduce stunting by 40% in 2025; United Nations, 2015) and the Sustainable Development Goal (Sustainable Development Goal‐2, end all forms of malnutrition by 2030; Sustainable Development Goal‐United Nations, 2015). This study aimed to investigate the trends and factors associated with complementary feeding practices (i.e., the introduction of solid, semi‐solid, and soft foods, minimum dietary diversity [MDD], minimum meal frequency [MMF], and MAD) in Ethiopia from 2005 to 2016.

## 2 METHODS

### 2.1 Data sources

The study was conducted using the Ethiopia Demographic and Health Survey (EDHS) data for 2005 (*N* = 2,520), 2011 (*N* = 2,850), and 2016 (*N* = 2,864). The EDHS collected nationally representative data on maternal and child health indicators, including infant and young child feeding (IYCF) practices of children under 2 years of age. The EDHS used a two‐stage stratified cluster sampling technique to select households from each enumeration area (EA). In the first stage, clusters were selected from a list of EAs from the population and housing census in Ethiopia (Central Statistics Agency [CSA], 2008). In the second stage, households were randomly selected from each EA after the complete household listing was conducted. The response rate of the surveys for the women questionnaire (which were used to collect information on infant and child health and nutrition) ranged from 94.6% in 2016 to 95.6% in 2005. The detailed methodology of the surveys has been reported in the respective EDHS reports (CSA [Ethiopia] and ICF [Inner City Fund] International, 2012, 2016; CSA [Ethiopia] and ORC Macro, 2006).

### 2.2 Outcome variables

The study outcome variables included the introduction of solid, semi‐solid, and soft foods, MDD, MMF, and MAD, measured according to mother's recall of foods given to the child during the day and at night prior to the survey (WHO & UNICEF, 2008).

1. Introduction of solid, semi‐solid, and soft foods (complementary foods) was defined as the proportion of infants 6–8 months of age who received solid, semi‐solid, or soft foods in the 24‐hr period, during the day and at night prior to the survey.
2. MDD was defined as the proportion of children 6–23 months of age who received foods from four or more of the seven food groups. The seven food groups included grains, roots, and tubers; legumes and nuts; dairy products (milk, yoghurt, and cheese); flesh foods (meat, fish, poultry, and liver/organ meats); eggs; vitamin A‐rich fruits and vegetables; and other fruits and vegetables.
3. MMF: For breastfed children, MMF was defined as the proportion of children who received solid, semi‐solid, or soft foods two times or more for children aged 6–8 months and three times or more for children aged 9–23 months over the 24‐hr period. For non‐breastfed children, MMF was defined as the proportion of children who received solid, semi‐solid, and soft foods plus other milk or milk products four times or more for children aged 6–23 months in the previous day.
4. MAD: For breastfed children, MAD was defined as the proportion of children 6–23 months of age who received MDD and MMF in the previous day (apart from breast milk). For non‐breastfed children, MAD was defined as the proportion of children 6–23 months of age who had received other milk or milk products and met the MDD (not including milk feeds) and the MMF in the day before.

### 2.3 Study variables

The study factors were selected based on the availability of information in the respective EDHS reports (CSA [Ethiopia] and ICF International, 2012, 2016; CSA [Ethiopia] and ORC Macro, 2006). Previous research conducted in Tanzania (Ogbo et al., 2018), Nigeria (Ogbo et al., 2015), Nepal (Na et al., 2018), Bangladesh (Na, Aguayo, Arimond, Narayan, & Stewart, 2018), and Afghanistan (Na, Aguayo, Arimond, Mustaphi, & Stewart, 2018) have also documented the importance of socioeconomic, demographic, health service, and community‐level factors associated with the introduction of solid, semi‐solid, and soft foods, MDD, MMF, and MAD. These variables informed the present study, and the variable categorization was based on the EDHS (CSA [Ethiopia] and ICF International, 2012, 2016; CSA [Ethiopia] and ORC Macro, 2006) and past studies (Na, Aguayo, Arimond, Dahal, et al., 2018; Na, Aguayo, Arimond, Narayan, & Stewart, 2018; Ogbo et al., 2018).

Socioeconomic factors included mother/father education, maternal occupation, and household wealth. Educational status was grouped as no schooling, primary education, or secondary education and higher. Mothers who were working in professional, technical, managerial, clerical, and services areas were grouped under formal occupation; those who were working in agriculture and manual works were grouped under informal occupation, and those who were not working were grouped under no occupation (Ahmed, Page, Arora, & Ogbo, 2019). The EDHS used principal components analysis to calculate the household wealth index based on a series of variables relating to ownership of household assets such as cattle and bicycles, type of materials used for housing construction, and types of water source and sanitation facilities. The EDHS categorized household wealth index into five quintiles (poorest, poorer, poor, rich, or richest). In this study, the household wealth index was re‐classified as “poor,” “middle,” or “rich” based on previously published studies (Ahmed et al., 2019; Ogbo et al., 2018) to increase the sample within each category.

Demographic factors included maternal age (grouped as 15–24 years, 25–34 years, or 35–49 years), sex of the child (male or female), birth order (categorized as 1, 2–4, or 5 and above), family size (categorized as ≤3, 4–5, or 6 and above members), desire for the pregnancy (grouped as desired or not desired), and media exposure (dichotomized as yes or no).

Health service factors included frequency of ANC visit (grouped as none, 1–4 visits, or 4 and above visits), place of birth (grouped as home or health facility delivery), and postnatal check‐up (grouped as yes or no).

Community‐level factors included place of residence (grouped as urban or rural) and region of residence (grouped as large central, small peripheral, and metropolis regions) based on their geopolitical features (Abrha, Shiferaw, & Ahmed, 2016; Ahmed et al., 2019). The large central region included Tigray, Amhara, Southern Nations Nationalities and Peoples Region, and Oromia regions, and the small peripheral region included Afar, Somali, Benishangul, and Gambella regions. The metropolis region included Addis Ababa and Dire Dawa city administrations and Harari region.

### 2.4 Statistical analysis

Initial analyses involved the calculation of the prevalence of the introduction of solid, semi‐solid, and soft foods, MDD, MMF, and MAD by each of the study factors (socioeconomic, demographic, health service, and community‐level factors) for each year of the EDHS to assess the extent to which the prevalence decreased or increased over the study period (2005–2016). Then, the percentage point change of the outcome variables (the introduction of complementary foods, MDD, MMF, and MAD) by each of the study factors was estimated in examining the changes over the EDHS years (from 2005 to 2011, from 2011 to 2016, and from 2005 to 2016; Tables S2, S3, S4, and S5).

A series of multivariate logistic regression models were used to investigate the association between the socioeconomic, demographic, health service, and community‐level factors with the introduction of solid, semi‐solid, and soft foods, MDD, MMF, and MAD. In Stage 1, the association between socioeconomic factors and the outcome variables was examined, while adjusting for demographic, health service, and community‐level confounding factors based on previously published studies (Issaka, Agho, Page, Burns, et al., 2015; Na, Aguayo, Arimond, Narayan, et al., 2018; Ogbo et al., 2015). In Stage 2, demographic factors were entered to the model to examine their relationship with the outcome measures, while adjusting for socioeconomic, health service, and community‐level confounders. In subsequent models (Stages 3 and 4), similar analytical strategies were used in examining the association between health service and community‐level factors and the outcome variables.

In this study, we used the combined data set to increase the statistical power of the study in order to detect any association between the study factors and the outcomes, as well as to examine trends in the complementary feeding indicators over the study period. In models of the combined data, a similar four‐stage analytical approach was used, as well as adjustment for year of the survey. We also estimated *p* for trend in models of the combined data to determine changes within each study factors over time. Odds ratios (ORs) with 95% confidence intervals (CIs) were calculated as the measure of association between the study factors and outcome variables. All analyses were conducted using Stata Version 14.0 with “svy” command for counts and percentages to adjust for sampling weights, clustering, and stratification effects; “lincom” command for estimating percentage points changes; and “melogit” function for multivariate models (StataCorp, 2017).

### 2.5 Ethics approval and consent to participate

The Ethiopia Demographic Health Survey (EDHS) was conducted after ethical approval was obtained from the National Research Ethics Review Committee (NRERC) in Ethiopia. During the survey, permission from administrative offices and verbal consent from study participants were obtained before the commencement of data collection. For this study, the data set was obtained after online submission of the proposal to MEASURE DHS/ICF website.

### 2.6 Availability of data and materials

The analysis was based on the data sets collected Ethiopian Demographic Health Survey. Information on the data and content can be accessed at https://dhsprogram.com/data/available-datasets.cfm

## 3 RESULTS

### 3.1 Characteristics of the study participants

Among the study participants, the majority (68.5%) of mothers had no schooling, and more than half (59.1%) of them had no occupation. Nearly 45% of mothers were resided in poor‐level households, and more than half of them had no ANC visit (Table S1).

### 3.2 Prevalence of complementary feeding practices by study factors

Over the study period, the highest prevalence of the introduction of solid, semi‐solid, and soft foods at 6–8 months of age was found among children whose mothers attended secondary or higher education (79.5%), whereas the lowest prevalence was observed among children who resided in the small peripheral region of Ethiopia (46.5%; Table 1). The proportion of MDD was highest among children whose mothers attended secondary or higher education (26.8%), whereas those who resided in the small peripheral region had the lowest prevalence of MDD (5.1%; Table 2). The highest prevalence of MMF (60.9%;Table 3) and MAD (17.8%; Table 4) was observed among children whose mothers attended secondary or higher education, whereas the lowest prevalence of MMF (33.2%; Table 3) and MAD (3.0%; Table 4) was among those who resided in the small peripheral region.

**Table 1:** Factors associated with the introduction of solid, semi‐solid, and soft foods in Ethiopia, 2005–2016

Variable	2005		2011		2016		2005–2016		2005–2016	*p* for trend
	*n* (%)	OR (95% CI)	*n* (%)	OR (95% CI)	*n* (%)	OR (95% CI)	*n* (%)	OR (95% CI)	% point change (95% CI)	
Socioeconomic factors^a^										
Maternal education										
No schooling	227 (49.4)	1.00	174 (41.8)	1.00	187 (59.0)	1.00	588 (49.3)	1.00	9.6 [−2.4, 21.6]	.741
Primary school	53 (49.4)	1.03 [0.54, 1.97]	108 (61.4)	2.18 [1.14, 4.15]	114 (54.1)	0.69 [0.41, 1.15]	276 (55.7)	1.17 [0.88, 1.57]	4.7 [−11.9, 21.2]	.909
Secondary and higher	21 (67.6)	2.28 [0.43, 11.94]	12 (75.7)	1.86 [0.48, 7.26]	39 (89.6)	2.76 [1.17, 6.49]	72 (79.5)	2.37 [1.34, 4.20]	22.0 [−2.7, 46.7]	.712
Maternal occupation										
No occupation	207 (47.5)	1.00	158 (43.0)	1.00	214 (60.2)	1.00	579 (50.0)	1.00	12.7 [1.6, 23.8]	.554
Formal occupation	24 (54.1)	0.83 [0.30, 2.25]	55 (74.4)	2.97 [1.42, 6.24]	42 (49.6)	0.74 [0.37, 1.49]	121 (59.6)	1.18 [0.81, 1.73]	−4.5 [−30.5, 21.6]	.648
Informal occupation	67 (58.3)	2.09 [1.00, 4.35]	77 (48.9)	1.78 [0.95, 3.35]	84 (63.8)	1.22 [0.71, 2.11]	228 (56.4)	1.45 [1.06, 1.99]	5.6 [−12.8, 23.9]	.225
Partner education										
No schooling	153 (52.4)	1.00	123 (43.4)	1.00	130 (55.7)	1.00	405 (50.2)	1.00	3.3 [−9.4, 15.9]	.900
Primary school	109 (46.5)	0.81 [0.47, 1.38]	135 (50.6)	1.5 [0.89, 2.54]	146 (59.9)	0.97 [0.6, 1.54]	389 (52.4)	1.10 [0.86, 1.42]	13.5 [0.4, 26.5]	.819
Secondary and higher	37 (56.7)	0.75 [0.31, 1.80]	37 (63.3)	1.45 [0.6, 3.52]	61 (67.5)	0.77 [0.41, 1.45]	135 (63.1)	0.96 [0.66, 1.40]	10.8 [−9.0, 30.7]	0.975
Household wealth status										
Poor	127 (51.7)	1.00	125 (46.1)	1.00	145 (56.5)	1.00	396 (51.3)	1.00	4.8 [−8.9, 18.5]	.889
Middle	70 (50.7)	1.09 [0.58, 2.06]	57 (47.9)	1.06 [0.52, 2.18]	91 (64.0)	1.83 [0.92, 3.61]	217 (54.6)	1.12 [0.81, 1.55]	13.3 [−2.6, 29.2]	.230
Rich	105 (48.5)	1.04 [0.56, 1.94]	112 (51.6)	0.73 [0.38, 1.43]	104 (60.3)	0.58 [0.29, 1.19]	322 (53.0)	0.76 [0.55, 1.04]	11.8 [−3.0, 26.5]	0.754
Demographic factors^b^										
Maternal age										
15–24 years	90 (52.6)	1.00	89 (47.5)	1.00	134 (64.2)	1.00	313 (55.2)	1.00	11.5 [−3.7, 26.8]	.527
25–34 years	138 (48.7)	0.69 [0.37, 1.31]	151 (52.5)	1.45 [0.81, 2.6]	152 (57.2)	0.82 [0.48, 1.40]	441 (52.7)	0.96 [0.71, 1.29]	8.6 [−3.0, 20.2]	.463
35–49 years	72 (50.9)	1.13 [0.45, 2.88]	55 (41.0)	1.32 [0.55, 3.15]	53 (55.5)	0.67 [0.28, 1.56]	181 (48.5)	1.05 [0.67, 1.65]	4.7 [−15.4, 24.7]	.118
Listening to radio										
No	192 (48.9)	1.00	131 (42.9)	1.00	231 (56.0)	1.00	554 (49.9)	1.00	71 [−3.7, 17.9]	.900
Yes	108 (53.1)	1.65 [0.93, 2.91]	164 (54.0)	1.25 [0.75, 2.09]	108 (68.6)	1.16 [0.67, 1.99]	380 (57.2)	1.15 [0.88, 1.49]	15.6 [1.7, 29.4]	.498
Reading newspaper/magazine										
No	274 (49.9)	1.00	264 (47.3)	1.00	308 (57.9)	1.00	845 (51.6)	1.00	8.0 [−1.5, 17.5]	.981
Yes	27 (54.9)	1.48 [0.52, 4.24]	30 (63.0)	1.97 [0.69, 5.63]	32 (81.1)	3.06 [1.11, 8.4]	89 (65.2)	1.50 [0.91, 2.49]	26.2 [2.2, 50.2]	.823
Watching TV										
No	269 (49.9)	1.00	181 (42.6)	1.00	263 (57.1)	1.00	713 (50.0)	1.00	7.3 [−2.4, 16.9]	.905
Yes	29 (52.6)	1.09 [0.39, 3.09]	111 (61.5)	1.27 [0.72, 2.25]	77 (69.1)	1.43 [0.75, 2.72]	217 (62.50)	1.19 [0.85, 1.68]	16.5 [−6.0, 39.0]	.377
Desire for the pregnancy										
Desired the pregnancy	251 (51.3)	1.00	266 (49.8)	1.00	310 (59.4)	1.00	827 (53.5)	1.00	8.0 [−1.2, 17.3]	.577
Not desired the pregnancy	50 (45.8)	0.67 [0.33, 1.35]	29 (38.5)	0.82 [0.35, 1.94]	29 (60.7)	0.75 [0.27, 2.11]	108 (46.6)	0.79 [0.53, 1.19]	14.9 [−10.8, 40.6]	.232
Health service factors^c^										
Antenatal visit										
None	202 (48.3)	1.00	162 (46.8)	1.00	99 (58.5)	1.00	462 (49.6)	1.00	10.2 [−4.6, 25.0]	.467
1–3	64 (57.9)	1.38 [0.75, 2.54]	69 (48.6)	1.27 [0.69, 2.33]	106 (58.7)	0.80 [0.47, 1.36]	238 (55.2)	1.11 [0.84,1.47]	0.8 [−16.8, 18.4]	.255
4+	35 (53.5)	1.68 [0.63, 4.5]	64 (53.7)	0.89 [0.41, 1.94]	125 (59.1)	0.76 [0.40, 1.43]	225 (56.6)	1.03 [0.71, 1.49]	5.7 [−13.2, 24.5]	.491
Postnatal check‐up										
No	276 (49.2)	1.00	286 (48.2)	1.00	306 (58.0)	1.00	868 (51.6)	1.00	8.8 [−0.1, 19.7]	.965
Yes	25 (67.6)	1.57 [0.55, 4.48]	9 (57.2)	2.07 [0.51, 1.67]	33 (77.5)	0.92 [0.39, 2.16]	67 (70.4)	1.36 [0.83, 2.22]	9.9 [−15.9, 35.7]	.814
Community‐level factors^d^										
Place of residence										
Urban	23 (56.1)	1.00	50 (55.0)	1.00	44 (70.7)	1.00	118 (60.2)	1.00	14.6 [−9.8, 39.0]	.855
Rural	277 (49.9)	0.99 [0.33, 2.99]	244 (47.3)	0.96 [0.37, 2.45]	296 (58.1)	0.85 [0.36, 1.97]	817 (51.7)	0.85 [0.54, 1.33]	8.2 [−1.2, 17.6]	.462
Region of residence										
Large central	278 (50.1)	1.00	271 (49.1)	1.00	301 (59.0)	1.00	850 (52.6)	1.00	8.9 [−0.7, 18.5]	.961
Small peripheral	16 (50.0)	1.01 [0.55, 1.86]	10 (30.9)	0.61 [0.35, 1.08]	25 (57.4)	0.93 [0.55, 1.6]	51 (46.5)	0.81 [0.61, 1.08]	9.5 [−6.3, 25.2]	.507
Metropolis	8 (66.9)	1.02 [0.43, 2.47]	13 (57.2)	1.07 [0.48, 2.38]	13 (79.8)	2.01 [0.9, 4.48]	34 (66.7)	1.08 [0.72, 1.63]	12.9 [−6.0, 31.9]	.102

*Note. n* (%): weighted count and proportion for each outcome variable by study factors; % point change indicates percentage point changes from 2005 to 2016.

Abbreviations: CI, confidence; OR, odds ratio.

a ORs of socioeconomic factors were adjusted for demographic, health service, and community‐level factors.

b ORs of demographic factors were adjusted for socioeconomic, health service, and community‐level factors.

c ORs of health service factors were adjusted for socioeconomic, demographic, and community‐level factors.

d ORs of community‐level factors were adjusted for socioeconomic, demographic, and health service factors.

**Table 2:** Factors associated with minimum dietary diversity in Ethiopia, 2005–2016

Variable	2005		2011		2016		2005–2016		2005–2016	*p* for trend
	*n* (%)	OR (95% CI)	*n* (%)	OR (95% CI)	*n* (%)	OR (95% CI)	*n* (%)	OR (95% CI)	% point change (95% CI)	
Socioeconomic factors										
Maternal education										
No schooling	96 (4.3)	1.00	45 (2.2)	1.00	189 (10.4)	1.00	330 (5.5)	1.00	6.1 [3.1, 9.1]	.078
Primary school	57 (11.3)	1.48 [0.88, 2.49]	69 (8.3)	1.84 [1.16, 2.92]	131 (14.1)	1.06 [0.77, 1.46]	256 (11.3)	1.42 [1.14, 1.77]	2.7 [−2.4, 7.9]	.320
Secondary and higher	27 (19.7)	1.57 [0.76, 3.24]	28 (21.7)	1.91 [0.94, 3.87]	82 (33.3)	1.71 [1.03, 2.85]	137 (26.8)	1.77 [1.27, 2.49]	13.7 [3.1, 24.3]	.002
Maternal occupation										
No occupation	122 (6.1)	1.00	59 (4.1)	1.00	212 (12.1)	1.00	393 (7.6)	1.00	6.0 [2.8, 9.1]	.004
Formal occupation	29 (12.7)	1.26 [0.74, 2.15]	39 (7.4)	1.47 [0.99, 2.20]	112 (23.7)	1.15 [0.79, 1.69]	180 (14.7)	1.23 [0.98, 1.56]	11.0 [3.0, 18.9]	.176
Informal occupation	29 (4.5)	1.01 [0.59, 1.75]	43 (4.4)	1.59 [0.97, 2.61]	78 (10.3)	1.36 [0.92, 2.01]	150 (6.4)	1.33 [1.03, 1.71]	5.8 [12.9, 10.3]	.777
Partner education										
No schooling	65 (4.1)	1.00	32 (2.3)	1.00	106 (8.6)	1.00	233 (5.3)	1.00	5.4 [2.2, 8.5]	.097
Primary school	58 (6.2)	1.52 [0.93, 2.48]	71 (5.7)	1.09 [0.69, 1.72]	172 (14.8)	1.37 [0.95, 1.97]	301 (9.0)	1.29 [1.02, 1.64]	8.6 [3.7, 13.4]	.151
Secondary and higher	56 (17.6)	1.64 [0.88,3.04]	35 (13.8)	1.52 [0.84, 2.72]	98 (24.1)	1.83 [1.23, 2.73]	189 (19.3)	1.65 [1.24, 2.19]	6.5 [−1.5, 14.5]	.002
Household wealth status										
Poor	50 (4.0)	1.00	40 (3.0)	1.00	120 (9.1)	1.00	211 (5.4)	1.00	5.0 [1.8, 8.3]	.567
Middle	28 (4.4)	1.06 [0.53, 2.12]	16 (2.7)	1.14 [0.57, 2.30]	81 (12.3)	1.73 [1.09, 2.77]	124 (6.6)	1.43 [1.03, 1.98]	7.9 [3.3, 12.5]	.046
Rich	102 (10.3)	1.46 [0.84, 2.52]	85 (8.4)	1.04 [0.54, 2.00]	201 (20.0)	1.78 [1.13, 2.79]	388 (12.9)	1.51 [1.11, 2.05]	9.6 [4.4, 14.8]	.003
Demographic factors										
Maternal age										
15–24 years	57 (6.9)	1.00	49 (5.7)	1.00	105 (12.5)	1.00	210 (8.3)	1.00	5.6 [1.4, 9.8]	.343
25–34 years	94 (6.8)	1.15 [0.72, 1.83]	77 (5.1)	1.20 [0.78, 1.84]	232 (15.1)	1.01 [0.69, 1.46]	403 (9.1)	1.12 [0.89, 1.41]	8.3 [4.4, 12.3]	.035
35–49 years	29 (4.5)	0.77 [0.38, 1.60]	15 (2.4)	1.11 [0.53, 2.31]	65 (10.7)	0.97 [0.58, 1.62]	109 (5.9)	0.99 [0.70, 1.38]	6.2 [1.6, 10.8]	.015
Listening to radio										
No	73 (4.0)	1.00	33 (2.2)	1.00	220 (10.2)	1.00	327 (5.9)	1.00	6.2 [3.6, 8.8]	.346
Yes	107 (10.5)	1.58 [0.96, 2.60]	108 (7.5)	1.37 [0.92, 2.03]	182 (22.3)	1.65 [1.18,2.29]	396 (12.1)	1.30 [1.05, 1.62]	11.8 [6.4, 17.2]	.002
Reading newspaper/magazine										
No	146 (5.5)	1.00	104 (3.9)	1.00	323 (11.6)	1.00	573 (7.0)	1.00	6.2 [3.5, 8.8]	.032
Yes	34 (18.5)	1.52 [0.81, 2.83]	37 (15.3)	1.32 [0.79, 2.19]	79 (36.9)	1.35 [0.87, 2.11]	150 (23.5)	1.27 [0.97, 1.66]	18.4 [6.3, 30.4]	.009
Watching TV										
No	131 (5.1)	1.00	39 (2.0)	1.00	263 (10.8)	1.00	433 (6.2)	1.00	5.7 [3.1, 8.3]	.126
Yes	49 (18.0)	2.00 [1.03, 3.88]	101 (10.3)	1.85 [1.17, 2.93]	139 (25.5)	0.97 [0.63, 1.50]	290 (16.1)	1.28 [0.98, 1.66]	7.5 [−1.9, 16.9]	.002
Desire for the pregnancy										
Desired the pregnancy	165 (7.0)	1.00	135 (5.1)	1.00	356 (13.0)	1.00	656 (8.5)	1.00	6.1 [3.2, 8.9]	.007
Not desired the pregnancy	15 (3.0)	1.12 [0.68, 1.83]	6 (2.0)	0.36 [0.13, 1.00]	46 (18.0)	1.52 [0.82, 2.81]	67 (6.4)	0.96 [0.69, 1.34]	14.9 [6.9, 23.0]	.163
Health service factors										
Antenatal visit										
None	110 (5.4)	1.00	42 (2.5)	1.00	125 (12.2)	1.00	277 (5.8)	1.00	6.8 [2.7, 10.9]	.569
1–3	18 (3.9)	0.73 [0.39, 1.36]	46 (6.4)	1.64 [0.96, 2.78]	101 (11.1)	1.13 [0.73, 1.75]	165 (7.9)	1.29 [0.98, 1.68]	7.1 [3.1, 11.2]	.002
4+	52 (14.3)	1.34 [0.72, 2.47]	53 (10.0)	2.21 [1.24, 3.95]	173 (16.8)	1.11 [0.71, 1.75]	278 (14.4)	1.56 [1.18, 2.07]	2.5 [−3.8, 8.7]	.057
Postnatal check‐up										
No	149 (5.5)	1.00	134 (4.7)	1.00	360 (13.1)	1.00	643 (7.7)	1.00	7.6 [4.8, 10.4]	.005
Yes	31 (19.2)	1.53 [0.81, 2.89]	7 (7.6)	1.86 [0.92, 3.77]	42 (17.3)	0.86 [0.55, 1.35]	80 (16.1)	1.12 [0.85, 1.48]	−1.9 [−12.1, 8.3]	.099
Community‐level factors										
Place of residence										
Urban	46 (21.2)	1.00	48 (12.0)	1.00	109 (30.1)	1.00	204 (20.7)	1.00	8.9 [−1.9, 19.7]	<.001
Rural	134 (50.1)	1.11 [0.50, 2.47]	92 (3.6)	0.76 [0.42, 1.38]	293 (11.2)	0.73 [0.45, 1.20]	519 (6.6)	0.91 [0.65, 1.27]	6.1 [3.4, 8.8]	.278
Region of residence										
Large central	164 (6.2)	1.00	124 (4.6)	1.00	352 (13.0)	1.00	639 (7.9)	1.00	6.8 [3.8, 9.9]	.217
Small peripheral	5 (3.1)	1.07 [0.60, 1.90]	7 (4.7)	1.54 [0.96, 2.46]	13 (7.1)	1.08 [0.72, 1.63]	25 (5.1)	1.13 [0.87, 1.47]	3.9 [0.9, 7.0]	.583
Metropolis	11 (18.6)	0.95 [0.51, 1.75]	10 (12.1)	1.09 [0.67, 1.76]	37 (37.3)	1.54 [1.03, 2.31]	58 (23.9)	1.10 [0.85, 1.42]	18.6 [6.7, 30.6]	.001

*Note. n* (%): weighted count and proportion for each outcome variable by study factors; % point change indicates percentage point changes from 2005 to 2016.

Abbreviations: CI, confidence; OR, odds ratio.

ORs of socioeconomic factors were adjusted for demographic, health service, and community‐level factors.

ORs of demographic factors were adjusted for socioeconomic, health service, and community‐level factors.

ORs of health service factors were adjusted for socioeconomic, demographic, and community‐level factors.

ORs of community‐level factors were adjusted for socioeconomic, demographic, and health service factors.

**Table 3:** Factors associated with minimum meal frequency in Ethiopia, 2005–2016

Variable	2005		2011		2016		2005–2016		2005–2016	*p* for trend
	*n* (%)	OR (95% CI)	*n* (%)	OR (95% CI)	*n* (%)	OR (95% CI)	*n* (%)	OR (95% CI)	% point change (95% CI)	
Socioeconomic factors										
Maternal education										
No schooling	869 (39.0)	1.00	875 (43.9)	1.00	764 (42.2)	1.00	2508 (41.6)	1.00	3.2 [−2.0, 8.5]	.386
Primary school	235 (47.1)	1.24 [0.94, 1.64]	469 (56.6)	1.31 [1.05, 1.65]	391 (41.9)	1.13 [0.89, 1.45]	1094 (48.4)	1.18 [1.04, 1.35]	−5.2 [−13.1, 2.7]	.191
Secondary and higher	80 (58.3)	1.37 [0.81, 2.29]	84 (66.3)	1.20 [0.74, 1.95]	147 (59.6)	2.05 [1.37, 3.08]	312 (60.9)	1.57 [1.22, 2.01]	1.3 [−12.5, 15.1]	.480
Maternal occupation										
No occupation	777 (38.9)	1.00	633 (44.2)	1.00	709 (40.5)	1.00	2119 (40.9)	1.00	1.6 [−3.6, 6.7]	.824
Formal occupation	131 (56.7)	1.23 [0.89, 1.68]	308 (59.3)	1.60 [1.26, 2.02]	211 (44.6)	1.11 [0.85, 1.46]	650 (53.1)	1.35 [1.16, 1.56]	−12.1[−23.9, −0.4]	.959
Informal occupation	274 (43.3)	1.16 [0.89, 1.51]	479 (49.2)	1.41 [1.13, 1.75]	381 (50.1)	1.63 [1.28, 2.08]	1134 (47.9)	1.43 [1.26, 1.63]	6.9 [−1.9, 15.7]	.147
Partner education										
No schooling	614 (38.6)	1.00	635 (44.9)	1.00	518 (41.8)	1.00	1841 (41.6)	1.00	2.9 [−2.6, 8.4]	.237
Primary school	393 (41.9)	1.11 [0.87, 1.41]	626 (50.4)	1.08 [0.89, 1.29]	491 (42.2)	0.82 [0.66, 1.02]	1509 (45.2)	1.03 [0.91, 1.15]	0.3 [−6.3, 6.9]	.270
Secondary and higher	171 (53.1)	1.18 [0.82, 1.71]	147 (58.1)	1.05 [0.76, 1.45]	225 (55.2)	0.99 [0.75, 1.31]	542 (55.3)	1.09 [0.91, 1.3]	2.1 [−8.0, 12.1]	.491
Household wealth status										
Poor	468 (37.3)	1.00	601 (44.5)	1.00	512 (38.7)	1.00	1581 (40.2)	1.00	1.4 [−4.4, 7.3]	.588
Middle	250 (40.1)	0.99 [0.75, 1.29]	305 (51.3)	1.18 [0.93, 1.50]	310 (47.3)	1.34 [0.99, 1.80]	865 (46.2)	1.13 [0.97, 1.31]	7.2 [−1.7, −16.2]	.216
Rich	467 (47.3)	1.19 [0.92, 1.53]	523 (52.0)	1.06 [0.84, 1.33]	479 (47.5)	1.01 [0.76, 1.35]	1468 (44.5)	1.11 [0.96, 1.28]	0.2 [−6.2, 6.6]	.731
Demographic factors										
Maternal age										
15–24 years	335 (40.3)	1.00	400 (46.7)	1.00	343 (40.9)	1.00	1078 (42.7)	1.00	0.6 [−6.3, 7.4]	.377
25–34 years	560 (40.2)	1.00 [0.78, 1.29]	752 (49.9)	1.18 [0.93, 1.50]	697 (45.4)	1.26 [0.98, 1.63]	2009 (45.3)	1.13 [0.99, 1.29]	5.2 [−0.01, 10.4]	.289
35–49 years	290 (45.1)	1.29 [0.91, 1.82]	276 (47.2)	1.08 [0.77, 1.52]	262 (42.7)	1.59 [1.11, 2.26]	828 (45.0)	1.27 [1.05, 1.53]	−2.5 [−10.4, 5.4]	.526
Listening to radio										
No	692 (37.4)	1.00	639 (42.6)	1.00	909 (41.8)	1.00	2240 (40.6)	1.00	4.4 [−0.2, 9.0]	.929
Yes	493 (48.5)	1.29 [1.04, 1.59]	789 (54.6)	1.36 [1.13, 1.65]	393 (48.2)	1.10 [0.87, 1.39]	1674 (51.1)	1.28 [1.14, 1.44]	−0.3 [−7.6, 7.0]	.719
Reading newspaper/magazine										
No	1093 (40.8)	1.00	1280 (47.3)	1.00	1192 (43.0)	1.00	3564 (43.7)	1.00	2.2 [−2.1, 6.5]	.576
Yes	89 (49.0)	1.89 [0.78, 1.80]	149 (61.7)	1.39 [0.95, 2.03]	110 (51.1)	0.87 [0.60, 1.28]	348 (54.5)	1.07 [0.87, 1.32]	2.0 [−10.7, 14.8]	.971
Watching TV										
No	1048 (40.5)	1.00	885 (45.0)	1.00	1010 (41.4)	1.00	2942 (42.1)	1.00	0.9 [−3.5, 5.3]	.660
Yes	134 (49.2)	0.76 [0.53, 1.09]	541 (55.1)	1.02 [0.83, 1.26]	291 (53.3)	1.19 [0.88, 1.60]	967 (53.7)	1.06 [0.91, 1.23]	4.1 [−6.3, 14.6]	.820
Desire for the pregnancy										
Desired the pregnancy	959 (40.7)	1.00	1316 (49.5)	1.00	1186 (43.4)	1.00	3461 (44.7)	1.00	2.7 [−1.6, 7.0]	.414
Not desired the pregnancy	226 (44.6)	1.24 [0.96, 1.59]	113 (38.4)	0.63 [0.45, 0.89]	116 (45.3)	1.08 [0.74, 1.60]	454 (43.1)	0.99 [0.83, 1.17]	7.6 [−10.6, 12.1]	.546
Health service factors										
Antenatal visit										
None	809 (39.9)	1.00	736 (43.6)	1.00	410 (40.1)	1.00	1956 (41.2)	1.00	−0.3 [−6.0, 6.6]	.280
1–3	181 (39.7)	0.80 [0.62, 1.05]	376 (52.2)	1.19 [0.97, 1.46]	403 (44.1)	0.89 [0.69, 1.15]	961 (45.9)	1.03 [0.91, 1.17]	4.5 [−3.4, 12.3]	.332
4+	187 (51.9)	1.31 [0.95, 1.81]	315 (59.1)	1.25 [0.96, 1.64]	477 (46.2)	0.91 [0.69, 1.19]	979 (50.8)	1.11 [0.95, 1.30]	−5.7 [−14.1, 2.8]	.111
Postnatal check‐up										
No	1095 (40.5)	1.00	1378 (48.2)	1.00	1189 (43.3)	1.00	3662 (44.1)	1.00	2.8 [−1.5, 7.1]	.662
Yes	89 (54.9)	1.14 [0.80, 1.63]	51 (54.7)	1.75 [1.15, 2.67]	113 (46.8)	1.15 [0.83, 1.60]	252 (50.9)	1.17 [0.97, 1.42]	−8.2 [−20.9, 4.6]	.522
Community‐level factors										
Place of residence										
Urban	115 (52.6)	1.00	210 (52.0)	1.00	203 (56.0)	1.00	529 (53.6)	1.00	3.4 [−7.0, 13.8]	.789
Rural	1069 (40.4)	1.00 [0.66, 1.52]	1218 (47.9)	1.16 [0.80, 1.66]	1098 (41.9)	0.82 [0.56, 1.21]	3386 (43.3)	0.95 [0.77, 1.18]	1.4 [−2.9, 5.8]	.458
Region of residence										
Large central	1101 (41.7)	1.00	1340 (49.2)	1.00	1165 (43.2)	1.00	3606 (44.7)	1.00	1.5 [−2.9, 6.0]	.305
Small peripheral	46 (28.1)	0.72 [0.54, 0.95]	42 (29.3)	0.54 [0.43, 0.67]	77 (40.5)	0.92 [0.72, 1.19]	165 (33.2)	0.74 [0.64, 0.84]	12.4 [4.9, 19.8]	.704
Metropolis	37 (61.8)	1.11 [0.80, 1.54]	47 (55.3)	0.98 [0.72, 1.33]	59 (59.3)	1.08 [0.79, 1.47]	143 (58.5)	1.04 [0.88, 1.24]	2.4 [−14.2, 9.3]	.915

*Note. n* (%): weighted count and proportion for each outcome variable by study factors; % point change indicates percentage point changes from 2005 to 2016.

Abbreviations: CI, confidence; OR, odds ratio.

ORs of socioeconomic factors were adjusted for demographic, health service, and community‐level factors.

ORs of demographic factors were adjusted for socioeconomic, health service, and community‐level factors.

ORs of health service factors were adjusted for socioeconomic, demographic, and community‐level factors.

ORs of community‐level factors were adjusted for socioeconomic, demographic, and health service factors.

**Table 4:** Factors associated with minimum acceptable diet in Ethiopia, 2005–2016

Variable	2005		2011		2016		2005–2016		2005–2016	*p* for trend
	*n* (%)	OR (95% CI)	*n* (%)	OR (95% CI)	*n* (%)	OR (95% CI)	*n* (%)	OR (95% CI)	% point change (95% CI)	
Socioeconomic factors										
Maternal education										
No schooling	60 (2.7)	1.00	40 (2.0)	1.00	94 (5.2)	1.00	194 (3.2)	1.00	2.6 [4.4, 4.7]	.053
Primary school	42 (8.4)	1.89 [0.95, 3.73]	58 (7.0)	1.92 [1.12, 3.29]	64 (6.8)	0.92 [0.60, 1.41]	164 (7.3)	1.41 [1.04, 1.89]	−1.6 [−5.5, 2.3]	.852
Secondary and higher	15 (11.3)	1.47 [0.62, 3.47]	22 (17.5)	1.99 [0.85, 4.66]	53 (21.6)	1.47 [0.77, 2.83]	91 (17.8)	1.68 [1.09, 2.59]	10.3 [−0.5, 21.1]	.049
Maternal occupation										
No occupation	74 (3.7)	1.00	51 (3.5)	1.00	106 (6.0)	1.00	230 (4.4)	1.00	2.3 [1.9, 4.5]	.034
Formal occupation	20 (8.8)	1.31 [0.72, 2.38]	31 (6.0)	1.33 [0.81, 2.17]	69 (14.5)	1.10 [0.67, 1.79]	120 (9.8)	1.23 [0.94, 1.62]	5.7 9 [−1.6, 13.1]	.427
Informal occupation	23 (3.7)	1.10 [0.60, 2.01]	38 (3.9)	1.43 [0.85, 2.42]	37 (4.9)	1.66 [1.06, 2.58]	99 (4.2)	1.47 [1.11, 1.94]	1.2 [−1.9, 4.4]	.488
Partner education										
No schooling	41 (2.6)	1.00	29 (2.1)	1.00	50 (4.0)	1.00	133 (3.0)	1.00	1.7 [−0.6, 4.0]	.551
Primary school	41 (4.3)	1.41 [0.79, 2.53]	62 (5.0)	1.09 [0.66, 1.82]	82 (7.0)	1.85 [1.15, 2.98]	184 (5.5)	1.46 [1.09, 1.96]	2.7 [−0.4, 5.8]	.244
Secondary and higher	35 (11.0)	1.83 [0.90, 3.73]	26 (10.3)	1.31 [0.68, 2.51]	71 (17.3)	2.87 [1.70, 4.85]	132 (13.4)	2.01 [1.42, 2.86]	6.3 [−1.2, 13.8]	.005
Household wealth status										
Poor	35 (2.8)	1.00	33 (2.4)	1.00	62 (4.7)	1.00	129 (3.3)	1.00	1.9 [−0.4, 4.2]	.971
Middle	17 (2.7)	0.74 [0.31, 1.74]	16 (2.7)	1.39 [0.64, 3.01]	54 (8.2)	1.94 [1.07, 3.52]	87 (4.6)	1.43 [0.96, 2.14]	5.5 [1.7, 9.3]	.021
Rich	66 (6.7)	1.39 [0.70, 2.77]	71 (7.1)	1.16 [0.57, 2.38]	96 (9.6)	1.66 [0.93, 2.95]	233 (7.8)	1.52 [1.05, 2.19]	2.9 [−0.1, 6.6]	.052
Demographic factors										
Maternal age										
15–24 years	39 (4.7)	1.00	46 (5.4)	1.00	57 (6.9)	1.00	142 (5.6)	1.00	2.2 [−1.2, 5.6]	.438
25–34 years	60 (4.3)	1.30 [0.72, 2.33]	61 (4.1)	1.05 [0.66, 1.68]	127 (8.3)	1.04 [0.64, 1.69]	248 (5.6)	1.09 [0.82, 1.45]	4.0 [1.1, 6.9]	.204
35–49 years	18 (2.9)	1.36 [0.53, 3.48]	13 (2.2)	0.94 [0.43, 2.04]	27 (4.4)	1.09 [0.58, 2.05]	58 (3.2)	1.11 [0.74, 1.68]	1.5 [−1.5, 4.5]	.032
Listening to radio										
No	47 (2.5)	1.00	28 (1.9)	1.00	124 (5.7)	1.00	199 (3.6)	1.00	3.2 [1.3, 5.1]	.349
Yes	71 (7.0)	1.65 [0.92, 2.96]	92 (6.3)	2.02 [1.27, 3.20]	89 (10.8)	1.47 [0.96, 2.25]	250 (7.6)	1.45 [1.12, 1.89]	3.8 [−0.4, 8.0]	.055
Reading newspaper/magazine										
No	96 (3.6)	1.00	92 (3.4)	1.00	167 (6.0)	1.00	355 (4.4)	1.00	2.5 [0.6, 4.4]	.055
Yes	21 (11.8)	1.77 [0.84, 3.71]	28 (11.5)	1.23 [0.71, 2.12]	44 (20.6)	1.14 [0.69, 1.88]	93 (14.7)	1.20 [0.87, 1.64]	8.8 [−2.0, 19.7]	.082
Watching TV										
No	93 (3.6)	1.00	36 (1.8)	1.00	135 (5.5)	1.00	263 (3.8)	1.00	1.9 [0.2, 3.7]	.296
Yes	24 (9.0)	0.89 [0.40, 2.03]	84 (8.6)	1.63 [0.99, 2.70]	76 (14.0)	0.89 [0.54, 1.46]	185 (10.3)	1.13 [0.83, 1.54]	5.0 [−2.1, 12.2]	.017
Desire for the pregnancy										
Desired the pregnancy	104 (4.4)	1.00	114 (4.3)	1.00	187 (6.8)	1.00	405 (5.2)	1.00	2.4 [0.4, 4.4]	.015
Not desired the pregnancy	13 (2.6)	1.27 [0.73, 2.18]	6 (2.0)	0.52 [0.19, 1.45]	25 (9.8)	1.10 [0.52, 2.35]	44 (4.2)	0.91 [0.61, 1.35]	7.2 [1.3, 13.2]	.744
Health service factors										
Antenatal visit										
None	809 (39.9)	1.00	37 (2.2)	1.00	70 (6.8)	1.00	174 (3.7)	1.00	3.5 [0.5, 6.4]	.386
1–3	181 (39.7)	0.66 [0.31, 1.42]	41 (5.8)	1.64 [0.88, 3.07]	49 (5.3)	0.95 [0.52, 1.73]	104 (5.0)	1.28 [0.92, 1.79]	2.3 [−0.7, 5.3]	.046
4+	187 (51.9)	1.64 [0.78, 3.43]	42 (7.9)	1.96 [1.00, 3.85]	93 (9.0)	0.91 [0.49, 1.69]	170 (8.8)	1.49 [1.03, 2.13]	−0.6 [−5.8, 4.6]	.209
Postnatal check‐up										
No	98 (3.6)	1.00	114 (4.0)	1.00	189 (6.9)	1.00	401 (4.8)	1.00	3.3 [1.3, 5.2]	.018
Yes	19 (11.5)	1.06 [1.09, 3.87]	6 (6.8)	1.47 [0.64, 3.40]	22 (9.2)	0.95 [0.57, 1.60]	47 (9.5)	1.14 [0.84, 1.56]	−2.4 [−10.4, 5.7]	.341
Community‐level factors										
Place of residence										
Urban	24 (11.0)	1.00	37 (9.2)	1.00	66 (18.2)	1.00	128 (12.9)	1.00	7.2 [−1.0, 15.5]	.007
Rural	93 (3.5)	1.61 [0.57, 4.53]	83 (3.3)	0.76 [0.41, 1.41]	145 (5.5)	0.66 [0.36, 1.21]	321 (4.1)	0.92 [0.63, 1.35]	2.0 [0.2, 3.9]	.536
Region of residence										
Large central	109 (4.1)	1.00	111 (4.1)	1.00	181 (6.7)	1.00	400 (5.0)	1.00	2.6 [0.4, 4.7]	.961
Small peripheral	2 (1.1)	0.66 [0.32, 1.37]	4 (3.0)	1.07 [0.63, 1.82]	9 (4.7)	1.51 [0.93, 2.46]	15 (3.0)	1.17 [0.87, 1.58]	3.6 [1.7, 5.4]	.507
Metropolis	6 (10.7)	1.05 [0.50, 2.17]	5 (6.1)	0.75 [0.45, 1.23]	22 (22.1)	1.33 [0.81, 2.19]	33 (13.7)	0.95 [0.70, 1.30]	11.5 [1.1, 21.8]	.102

*Note. n* (%): weighted count and proportion for each outcome variable by study factors; % point change indicates percentage point changes from 2005 to 2016.

Abbreviations: CI, confidence; OR, odds ratio.

ORs of socioeconomic factors were adjusted for demographic, health service, and community‐level factors.

ORs of demographic factors were adjusted for socioeconomic, health service, and community‐level factors.

ORs of health service factors were adjusted for socioeconomic, demographic, and community‐level factors.

ORs of community‐level factors were adjusted for socioeconomic, demographic, and health service factors.

### 3.3 Trends in complementary feeding practices

The proportion of children who met MDD significantly increased from 6.3% (95% CI: 5.1, 7.8%) in 2005 to 13.5% (95% CI: 11.3, 16.0%) in 2016 (*p* trend < .001), and MAD also significantly increased from 4.1% (95% CI: 3.2, 5.3%) in 2005 to 7.1% (95% CI: 5.6, 8.9%) in 2016 (*p* trend = .003). Between 2005 to 2016, the highest increase in the prevalence of introduction of complementary foods was observed among children whose mothers read magazine/newspaper (percentage point change = 26.2%; 95% CI: 2.2, 50.2%; Table 1). The largest percentage point increase in MDD was found among those who resided in the metropolis region (percentage point change = 18.6%; 95% CI: 6.7, 30.6%; Table 2).

**Figure 1:**
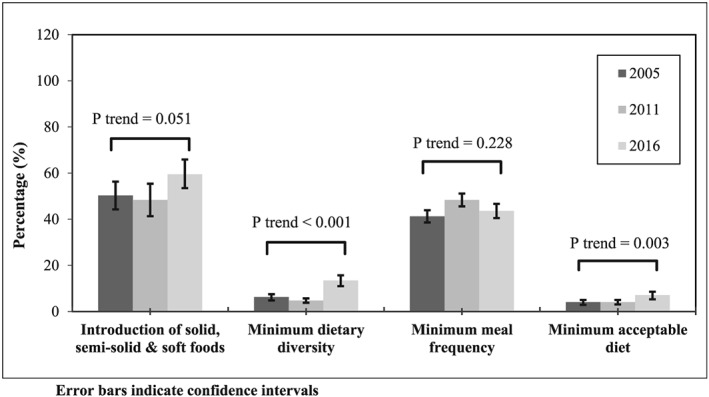
Prevalence and trends of complementary feeding practices in Ethiopia, 2005–2016

The prevalence of the introduction of complementary foods increased from 50.3% (95% CI: 44.3, 56.3%) in 2005 to 59.5% (95% CI: 53.1, 65.5%) in 2016 (*p* trend = .051), and MMF increased from 41.3% (95% CI: 38.7, 44.0%) in 2005 to 43.6% (95% CI: 40.5, 46.7%) in 2016 (*p* trend = 0.228), but the increases were not statistically significant (Figure 1). Between 2005 to 2016, children who resided in the small peripheral region showed the highest percentage increase change in MMF (percentage point change = 12.4%; 95% CI: 4.9, 19.8%; Table 3), and those who resided in the metropolis region had the highest percentage point increase in MAD (percentage point change = 11.5%; 95% CI: 1.1, 21.8%; Table 4).

### 3.4 Factors associated with the introduction of complementary foods

From 2005 to 2016, infants whose mothers attended secondary or higher education were more likely to introduce solid, semi‐solid, and soft foods at 6–8 months of age compared with those who had no schooling (OR = 2.37; 95% CI [1.34, 4.40]; Table 1). The likelihood of the introduction of complementary foods was significantly higher among infants whose mothers who had informal occupations compared with those who had no occupation (OR = 1.45; 95% CI [1.07, 1.99]; Table 1).

### 3.5 Factors associated with MDD

Over the study period, the odds of MDD were significantly higher in children whose mothers or fathers attended secondary or higher education compared with those who did not attend formal schooling (OR = 1.77; 95% CI [1.27, 2.49] for mothers and OR = 1.65; 95% CI [1.24, 2.19] for fathers; Table 2). Children whose mothers resided in wealthy households had higher odds of MDD compared with those who were from poor households (OR = 1.51; 95% CI [1.11, 2.05]). Children whose mothers had four or more ANC visits were more likely to meet MDD compared with those who had no ANC visits (OR = 1.56; 95% CI [1.18, 2.07]). Children whose mothers listened to the radio had higher odds of MDD compared with those who did not listen to the radio (OR = 1.30; 95% CI [1.05, 1.62]; Table 2).

### 3.6 Factors associated with MMF

Over the study period, children whose mothers attended secondary or higher education (OR = 1.57; 95% CI: 1.22, 2.01), those employed in informal jobs (OR = 1.35; 95% CI: 1.16, 1.56) or formal jobs (OR = 1.43; 95% CI: 1.26, 1.63) and those who listened to the radio (OR = 1.28; 95% CI: 1.14, 1.44) had higher odds of MMF compared with their counterparts. The likelihood of MMF was lower among children who resided in the small peripheral region (i.e. Afar, Somali, Benishangul and Gambella regions) compared with children whose mothers who resided in the larger central region (OR = 0.74; 95% CI: 0.64, 0.84) [Table 3].

### 3.7 Factors associated with MAD

Over the study period, children who resided in wealthy households had a higher odds of MAD compared with those who were from poor households (OR = 1.47; 95% CI [1.11, 1.94]). Children whose fathers attended secondary or higher education were more likely to meet MAD compared with those whose fathers had no schooling (OR = 2.01; 95% CI [1.42, 2.86]). Children whose mothers had frequent ANC (≥4) visits had higher odds of MAD compared with those who had no ANC visits (OR = 1.49; 95% CI [1.04, 2.13]). The likelihood of MAD was higher among children whose mothers listened to the radio compared with their counterparts (OR = 1.45; 95% CI [1.12, 1.89] Table 4).

## 4 DISCUSSION

The present study showed improvements in the proportion of children who met MDD (from 6.3% to 13.5%) and MAD (from 4.1% to 7.1%) between 2005 and 2016. There were also improvements in the introduction of solid, semi‐solid, and soft foods (from 50.3% to 59.5%) and MMF (from 41.3% to 43.6%), but were not statistically significant. Improvements in the MDD and MAD may reflect the impact of the national IYCF strategy and any other nutrition initiative in Ethiopia (Ethiopian Federal Ministry of Health, 2004). However, the progress reported in some complementary feeding indicators has not kept pace with the overall economical growths reported in the last two decades in Ethiopia (The World Bank, 2019). Scaling up current national nutritional efforts on food adequacy and dietary diversity would possibly be helpful to Ethiopian mothers to increase IYCF practices.

Over the study period, higher maternal education and occupation were associated with the introduction of complementary foods, MDD, MMF, and MAD. Higher partner education, frequent ANC (≥4) visits, and those who resided in wealthy households were associated with MDD and MAD. Children whose mothers listened to the radio had higher odds of MDD, MMF, and MAD compared with those who did not listen to the radio. Children of mothers who resided in the small peripheral region (i.e., Afar, Somali, Benishangul, and Gambella regions) were less likely to meet MMF compared with those who resided in the large central region. Additionally, maternal occupation and listening to the radio were the most common factors associated with higher odds of MMF over the study period.

Research from many LMICs showed that parental education, particularly maternal education, has a significant impact on the nutritional status of infants and young children (Alderman & Headey, 2017; Iftikhar, Bari, Bano, & Masood, 2017; Smith‐Greenaway, 2013). The present study also indicated that higher maternal education was associated with the introduction of solid, semi‐solid, and soft foods, MDD, MMF, and MAD, consistent with national studies conducted in Tanzania (Ogbo et al., 2018), Nigeria (Ogbo et al., 2015), Sri Lanka (Senarath, Godakandage, Jayawickrama, Siriwardena, & Dibley, 2012), India (Patel et al., 2012), and Bangladesh (Kabir et al., 2012). A possible explanation for the observed relationship could be that education helps the mother to improve her knowledge on healthy eating behaviour for infant and young children such as attentive child feeding practices and providing an appropriately diversified diet (Alderman & Headey, 2017; Pan American Health Organization & WHO, 2001). Higher maternal education may also create the pathway for better access and benefit from child feeding programs (Alderman & Headey, 2017; Black et al., 2013; Guldan et al., 1993). In addition, available feeding programs (often delivered as written materials, pamphlets, or books) might not be suitable for mothers with no schooling (Fein, Labiner‐Wolfe, Scanlon, & Grummer‐Strawn, 2008).

The study also found that children with educated fathers were more likely to meet MDD and MAD, indicating the positive impact of fathers' education on complementary feeding practices. The relationship between mothers' and fathers' education with complementary feeding practices indicates that achieving universal primary education in adolescent girls (future mothers) would be helpful in improving the nutritional status of infant and young children in Ethiopia.

Our study showed that mothers who were in occupation were more likely to timely introduce solid, semi‐solid, and soft foods to their infants. Similarly, infants whose mothers were in occupation were more likely to meet MDD, MMF, and MAD. These findings are consistent with nationally representative studies conducted in Tanzania (Ogbo et al., 2018), Nigeria (Ogbo et al., 2015), Nepal (Joshi, Agho, Dibley, Senarath, & Tiwari, 2012), and India (Patel et al., 2012). According to previous studies, maternal occupation has two conflicting relationships with the feeding of infant and young children. First, maternal occupation may increase household income with subsequent empowerment of women and improvement in the dietary intake of children (Eshete, Abebe, Loha, Gebru, & Tesheme, 2017; Tucker & Sanjur, 1988). Second, maternal occupation may reduce the time a mother can spend in caring and feeding of their children (Eshete et al., 2017; Nair, Ariana, & Webster, 2014; Tucker & Sanjur, 1988). In Ethiopia, policymakers and programme planners would do well to consider interventions that acknowledge these conflicting relationships between maternal occupation and complementary feeding. For example, arranging child day‐care centres near the working area of mothers may be helpful for children to receive attention and time and for the provision of diversified complementary foods (Hirani & Karmaliani, 2013).

Child nutrition education and counselling during ANC and postnatal care visits are also among the effective and less expensive strategies to improve the complementary feeding practice of mothers (WHO, 2009). The present study found that a higher frequency of ANC (≥4) visits was associated with meeting MDD and MMF compared with no ANC visits. This finding is consistent with similar studies conducted in Nigeria (Ogbo et al., 2015), India (Patel et al., 2012), Nepal (Na, Aguayo, Arimond, Dahal, et al., 2018), and Pakistan (Na, Aguayo, Arimond, & Stewart, 2017). ANC visit is often the entry point for mothers to receive appropriate nutritional education and counselling, with subsequent improvement in the mother's knowledge on IYCF (Tariku et al., 2017; Wu et al., 2014). Improving the mother's knowledge is also associated with a positive attitude and a good practice of complementary feeding for children (Blaney, Februhartanty, & Sukotjo, 2015). Strengthening the maternal health care system with nutritional counselling and education during the antenatal and postnatal visits would be helpful in improving complementary feeding practices in Ethiopia.

Children whose mothers listened to the radio had higher odds of meeting MDD, MMF, and MAD than their counterparts, and this finding is consistent with studies conducted in India (Patel et al., 2012) and Nepal (Joshi et al., 2012). In Ethiopia, in recent years, IYCF practices have received significant mass media coverage, which may explain the reason for the observed relationship between a mother's listening to the radio and optimal complementary feeding practices. This finding is also supported by research conducted in Bangladesh, which indicated that intensive counselling combined with nationwide mass media campaign increased the prevalence of MAD from 16% to 50% (Menon et al., 2016). The relationship between listening to the radio and complementary feeding practices can also be explained by listening to the radio being a proxy marker for household wealth and education. Our result suggests that a nationwide mass media campaign may be needed as they remain strategically important in improving IYCF practices.

The present study showed that children who resided in the small peripheral region (i.e., Afar, Somali, Benishangul, and Gambella) were less likely to meet the MMF compared with those who resided in the large central region. This finding may be due to economic reason as the large central region are more developed, with higher socioeconomic status as a result of their fertile soil and rainfall throughout the year. On the other hand, the latter regions were among the areas that suffered from repeated drought, with adverse effects on food production and availability (Food and Agriculture Organization, 2017).

### 4.1 Study limitations and strengths

The following limitations should be considered while interpreting the findings of this study: (a) cross‐sectional nature of the data might not allow inferring casual direction.; (b) social desirability bias as a result of the tendency to respond to socially desirable answers for dietary diversity and meal frequency questions may have occurred, which may result in overestimation of the proportion of MDD and MMF; (c) overestimation or underestimation of the ORs could also be expected as a result of misclassification bias in the grouping of the variables such as frequency of ANC visit and birth order; and (d) unmeasured confounding factors such as parents' interactions with each other and with their children, communication between family members, and the influence of grandmothers can also be considered as the limitation of this study. Despite the above limitation, nationally representativeness of the data and the high response rate of the surveys are the strengths of this study. The study was also based on EDHS that used a standard questionnaire, which allows comparability of the findings across time and regions.

## 5 CONCLUSION

The study indicates that MDD and MAD improved between 2005 and 2016; however, their levels remain unacceptably low. Over the same period, the introduction of complementary foods and MMF increased but were not statistically significant. Maternal education and occupation were the common factors associated with timely introduction of complementary foods, MDD, MMF, and MAD. Higher partner education and frequent antenatal visits were associated with MDD and MAD. Listening to the radio increased the odds of MDD, MMF, and MAD. National IYCF policy interventions that aim to improve complementary feeding practices of Ethiopian mothers should also focus on those with limited education, antenatal service usage, and media exposure.

## CONFLICTS OF INTEREST

The authors declare that they have no conflicts of interest.

## CONTRIBUTIONS

KYA contributed to the conception of the study, obtained and analysed the data, drafted the manuscript, interpreted the results, and critically revised the manuscript. AP and AA contributed to the conception of the idea and critically revised the manuscript. All authors read and approved the final manuscript. FAO contributed to the conception of the idea, drafting, analysis, and interpretation of the data and critically revised the manuscript.

## Supporting information

Table S1.
Characteristics of the study participants in Ethiopia, 2005–2016

Table S2.
Percentage point change in prevalence of solid, semi‐solid and soft foods by study factors, 2005–2016

Table S3.
Percentage point change in the prevalence of minimum dietary diversity by study factors, 2005–2016

Table S4.
Percentage point change in the prevalence of minimum meal frequency by study factors, 2005–2016

Table S5.
Percentage point change in the prevalence of minimum acceptable diet by study factors, 2005–2016
