# Supplementary material for: Trends and factors associated with complementary feeding practices in Ethiopia from 2005 to 2016
Source: Matern Child Nutr. 2019 Dec 12;16(2):e12926. doi: 10.1111/mcn.12926 (PMC7083482; doi:10.1111/mcn.12926)
Supplement: Supplementary file 1 — Table S1. Characteristics of the study participants in Ethiopia, 2005–2016 [file MCN-16-e12926-s001.docx]

**Table S1**

Characteristics of the study participants in Ethiopia, 2005–2016

| **Variables** | **2005**  **(N= 2520)** | **2011**  **(N= 2850)** | **2016**  **(N= 2864)** | **2005–2016**  **(N=8234)** |
| --- | --- | --- | --- | --- |
|  | **n(%)** | **n(%)** | **n(%)** | **n(%)** |
| Socioeconomic factors | | | | |
| Maternal education |  |  |  |  |
| No schooling | 2229 (77.8) | 1993 (67.6) | 1808 (60.5) | 6030 (68.5) |
| Primary school | 499 (17.4) | 829 (28.10 | 931 (31.2) | 2259 (25.7) |
| Secondary and higher | 137 (4.8) | 127 (4.3) | 247 (8.3) | 512 (5.8) |
| Maternal occupation |  |  |  |  |
| No occupation | 1997 (69.8) | 1432 (49.0) | 1754 (58.7) | 5182 (59.1) |
| Formal occupation | 231 (8.1) | 519 (17.7) | 473 (15.8) | 1223 (13.9) |
| Informal occupation | 633 (22.1) | 974 (33.3) | 760 (25.5) | 2367 (27.0) |
| Partner education |  |  |  |  |
| No schooling | 1584 (55.8) | 1415 (48.6) | 1239 (44.1) | 4427 (50.6) |
| Primary school | 936 (33.0) | 1243 (42.7) | 1162 (41.4) | 3340 (38.2) |
| Secondary and higher | 321 (11.3) | 253 (8.7) | 407 (14.5) | 981 (11.2) |
| Household wealth status |  |  |  |  |
| Poor | 1256 (43.8) | 1351 (45.8) | 1323 (44.3) | 3929 (44.6) |
| Middle | 623 (21.8) | 594 (20.1) | 656 (22.0) | 1873 (21.3) |
| Rich | 987 (34.4) | 1005 (34.1) | 1007 (33.7) | 2999 (34.1) |
| Demographic factors | | | | |
| Maternal age |  |  |  |  |
| 15-24 years | 831 (29.0) | 857 (29.0) | 839 (28.1) | 2526 (28.7) |
| 25-34 years | 1392 (48.6) | 1507 (51.1) | 1535 (51.4) | 4434 (50.4) |
| 35-49 years | 642 (22.4) | 586 (19.9) | 613 (20.5) | 1841 (20.9) |
| Child sex |  |  |  |  |
| Male | 1455 (50.8) | 1497 (50.8) | 1402 (46.9) | 4355 (49.5) |
| Female | 1410 (49.2) | 1452 (49.2) | 1584 (53.1) | 4446 (50.5) |
| Birth order |  |  |  |  |
| One | 483 (16.8) | 506 (17.2) | 580 (19.4) | 1569 (17.8) |
| 2-4 | 1228 (42.8) | 1349 (45.7) | 1293 (43.3) | 3870 (44.0) |
| 5+ | 1155 (40.3) | 1094 (37.1) | 1114 (37.3) | 3363 (38.2) |
| Listening radio |  |  |  |  |
| No | 1850 (64.6) | 1502 (51.0) | 2172 (72.7) | 5523 (62.8) |
| Yes | 1016 (34.5) | 1445 (49.0) | 815 (27.3) | 3276 (37.2) |
| Reading newspaper/magazine |  |  |  |  |
| No | 2680 (93.6) | 2706 (91.8) | 2771 (92.8) | 8158 (92.7)) |
| Yes | 182 (6.4) | 241 (8.2) | 215 (7.2) | 638 (7.3) |
| Watching TV |  |  |  |  |
| No | 2587 (90.5) | 1964 (66.7) | 2440 (81.7) | 6992 (79.5) |
| Yes | 273 (9.5) | 982 (33.3) | 546 (18.3) | 1801 (20.5) |
| Wanted pregnancy |  |  |  |  |
| Then/later | 2358 (82.3) | 2656 ( 90.1) | 2732 (91.5) | 7746 (88.0) |
| No more | 506 (17.7) | 293 (9.9) | 255 (8.5) | 1054 (12.0) |
| Health service factors | | | | |
| Antenatal Visit |  |  |  |  |
| None | 2030 (71.3) | 1689 (57.4) | 1022 (34.4) | 4742 (54.1) |
| 1-3 | 457 (16.0) | 720 (24.5) | 914 (30.8) | 2091 (23.9) |
| 4+ | 361 (12.7) | 532 (18.1) | 1032 (34.8) | 1925 (22.0) |
| Mode of delivery |  |  |  |  |
| Not caesarean | 2836 (99.0) | 2890 (98.0) | 2909 (97.4) | 8634 (98.1) |
| Caesarean | 29 (1.0) | 60 (2.0) | 77 (2.6) | 166 (1.9) |
| Place of birth |  |  |  |  |
| Home | 2686 (93.7) | 2613 (88.6) | 1903 (63.7) | 7202 (81.8) |
| Health facility | 180 (6.3) | 336 (11.4) | 1083 (36.3) | 1599 (18.2) |
| Delivery assistance |  |  |  |  |
| Health professional | 302 (11.2) | 348 (12.4) | 1107 (43.7) | 1757 (21.9) |
| Traditional birth attendant | 374 (13.9) | 175 (6.2) | 1004 (39.6) | 1553 (19.3) |
| Others untrained | 2014 (74.9) | 2291 (81.4) | 425 (16.8) | 4729 (58.8) |
| Postnatal check-up |  |  |  |  |
| No | 2703 (94.3) | 2856 (96.9) | 2746 (91.9) | 8305 (94.4) |
| Yes | 162 (5.7) | 93 (3.2) | 241 (8.1) | 496 (5.6) |
| Community-level factors | | | | |
| Place of residence |  |  |  |  |
| Urban | 219 (7.7) | 404 (1.7) | 363 (12.2) | 986 (11.2) |
| Rural | 2646 (92.4) | 2545 (86.3) | 2623 (87.8) | 7815 (88.8) |
| Region of residence |  |  |  |  |
| Large central | 2642 (92.2) | 2722 (92.3) | 2697 (90.3) | 8060 (91.6) |
| Small peripheral | 164 (5.7) | 143 (4.8) | 190 (6.4) | 497 (5.6) |
| Metropolis | 60 (2.1) | 85 (2.9) | 99 (3.3) | 244 (2.8) |

**n (%): weighted count and proportion of study factors**
