# Supplementary material for: Trends and factors associated with complementary feeding practices in Ethiopia from 2005 to 2016
Source: Matern Child Nutr. 2019 Dec 12;16(2):e12926. doi: 10.1111/mcn.12926 (PMC7083482; doi:10.1111/mcn.12926)
Supplement: Supplementary file 2 — Table S2. Percentage point change in prevalence of solid, semi‐solid and soft foods by study factors, 2005–2016 [file MCN-16-e12926-s002.docx]

**Table S2**

Percentage point change in prevalence of solid, semi-solid and soft foods by study factors, 2005–2016

| **Variables** | **2005** | **2005–2011** | **2011** | **2011–2016** | **2016** | **2005–2016** | **2005–2016** |
| --- | --- | --- | --- | --- | --- | --- | --- |
|  | **n (%)** | **Diff -1**  **(95% CI)** | **n (%)** | **Diff-2**  **(95% CI)** | **n (%)** | **Diff-3**  **(95% CI)** | **n (%)** |
| **Socioeconomic factors** |  |  |  |  |  |  |  |
| Maternal education |  |  |  |  |  |  |  |
| No schooling | 227 (49.4) | -7.6 (-17.9, 2.8) | 174 (41.8) | 17.2 (4.5, 29.9) | 187 (59.0) | 9.6 (-2.4, 21.6) | 588 (49.3) |
| Primary school | 53 (49.4) | 12.0 (-5.5, 29.5) | 108 (61.4) | -7.3 (-22.3, 7.7) | 114 (54.1) | 4.7 (-11.9, 21.2) | 276 (55.7) |
| Secondary and higher | 21 (67.6) | 8.2 (-21.2, 37.6) | 12 (75.7) | 13.9 (-8.4, 36.2) | 39 (89.6) | 22.0 (-2.7, 46.7) | 72 (79.5) |
| Maternal occupation |  |  |  |  |  |  |  |
| No occupation | 207 (47.5) | -4.6 (-16.1, 7.0) | 158 (43.0) | 17.3 (4.7, 29.8) | 214 (60.2) | 12.7 (1.6, 23.8) | 579 (50.0) |
| Formal occupation | 24 (54.1) | 20.4 (-4.4, 45.1) | 55 (74.4) | -24.8 (-45.7, -4.0) | 42 (49.6) | -4.5 (-30.5, 21.6) | 121 (59.6) |
| Informal occupation | 67 (58.3) | -9.4 (-26.6, 7.8) | 77 (48.9) | 15.0 (-2.3, 32.2) | 84 (63.8) | 5.6 (-12.8, 23.9) | 228 (56.4) |
| Partner education |  |  |  |  |  |  |  |
| No schooling | 153 (52.4) | -0.0 (-20.5, 2.6) | 123 (43.4) | 12.3 (-1.2, 25.7) | 130 (55.7) | 3.3 (-9.4, 15.9) | 405 (50.2) |
| Primary school | 109 (46.5) | 4.2 (-9.6, 18.0) | 135 (50.6) | 9.3 (-4.1, 22.7) | 146 (59.9) | 13.5 (0.4, 26.5) | 389 (52.4) |
| Secondary and higher | 37 (56.7) | 6.6 (-15.5, 28.8) | 37 (63.3) | 4.2 (-17.8, 26.2) | 61 (67.5) | 10.8 (-9.0, 30.7) | 135 (63.1) |
| Household wealth status |  |  |  |  |  |  |  |
| Poor | 127 (51.7) | -5.7 (-17.9, 6.5) | 125 (46.1) | 10.4 (-2.8, 23.7) | 145 (56.5) | 4.8 (-8.9, 18.5) | 396 (51.3) |
| Middle | 70 (50.7) | -2.8 (-21.7, 16.2) | 57 (47.9) | 16.0 (-2.3, 34.4) | 91 (64.0) | 13.3 (-2.6, 29.2) | 217 (54.6) |
| Rich | 105 (48.5) | 3.1 (-11.9, 18.0) | 112 (51.6) | 8.7 (-7.8, 25.1) | 104 (60.3) | 11.8 (-3.0, 26.5) | 322 (53.0) |
| **Demographic factors** |  |  |  |  |  |  |  |
| Maternal age |  |  |  |  |  |  |  |
| 15–24 years | 90 (52.6) | -5.1 (-20.6, 10.4) | 89 (47.5) | 16.7 (1.7, 31.7) | 134 (64.2) | 11.5 (-3.7, 26.8) | 313 (55.2) |
| 25–34 years | 138 (48.7) | 3.8 (-8.2, 15.9) | 151 (52.5) | 4.7 (-7.5, 17.0) | 152 (57.2) | 8.6 (-3.0, 20.2) | 441 (52.7) |
| 35–49 years | 72 (50.9) | -9.9 (-27.9, 8.2) | 55 (41.0) | 14.5 (-7.1, 36.1) | 53 (55.5) | 4.7 (-15.4, 24.7) | 181 (48.5) |
| Listening radio |  |  |  |  |  |  |  |
| No | 192 (48.9) | -6.0 (-17.6, 5.5) | 131 (42.9) | 13.1 (1.2, 25.0) | 231 (56.0) | 71 (-3.7, 17.9) | 554 (49.9) |
| Yes | 108 (53.1) | 0.9 (-12.3, 14.2) | 164 (54.0) | 14.6 (1.1, 28.2) | 108 (68.6) | 15.6 (1.7, 29.4) | 380 (57.2) |
| Reading newspaper/magazine |  |  |  |  |  |  |  |
| No | 274 (49.9) | -2.6 (-12.3, 7.1) | 264 (47.3) | 10.6 (0.5, 20.6) | 308 (57.9) | 8.0 (-1.5, 17.5) | 845 (51.6) |
| Yes | 27 (54.9) | 8.1 (-23.8, 39.9) | 30 (63.0) | 18.1 (-12.5, 48.7) | 32 (81.1) | 26.2 (2.2, 50.2) | 89 (65.2) |
| Watching TV |  |  |  |  |  |  |  |
| No | 269 (49.9) | -7.3 (-17.3, 2.7) | 181 (42.6) | 14.6 (3.7, 25.5) | 263 (57.1) | 7.3 (-2.4, 16.9) | 713 (50.0) |
| Yes | 29 (52.6) | 8.9 (-13.0, 30.8) | 111 (61.5) | 7.6 (-8.6, 23.8) | 77 (69.1) | 16.5 (-6.0, 39.0) | 217 (62.50 |
| Desire for the pregnancy |  |  |  |  |  |  |  |
| Desired the pregnancy | 251 (51.3) | -1.5 (-11.3, 8.2) | 266 (49.8) | 9.6 (0.0, 19.2) | 310 (59.4) | 8.0 (-1.2, 17.3) | 827 (53.5) |
| Not desired the pregnancy | 50 (45.8) | -7.3 (-29.6, 15.0) | 29 (38.5) | 22.2 (-6.8, 51.2) | 29 (60.7) | 14.9 (-10.8, 40.6) | 108 (46.6) |
| **Health service factors** |  |  |  |  |  |  |  |
| Antenatal Visit |  |  |  |  |  |  |  |
| None | 202 (48.3) | -1.5 (-12.8, 9.8) | 162 (46.8) | 11.7 (-4.1, 27.5) | 99 (58.5) | 10.2 (-4.6, 25.0) | 462 (49.6) |
| 1–3 | 64 (57.9) | -9.3 (-27.7, 9.1) | 69 (48.6) | 10.1 (-7.6, 27.7) | 106 (58.7) | 0.8 (-16.8, 18.4) | 238 (55.2) |
| 4+ | 35 (53.5) | 0.2 (-21.7, 22.2) | 64 (53.7) | 5.4 (-12.2, 23.1) | 125 (59.1) | 5.7 (-13.2, 24.5) | 225 (56.6) |
| Postnatal check-up |  |  |  |  |  |  |  |
| No | 276 (49.2) | -1.0 (-10.6, 8.5) | 286 (48.2) | 9.8 (-0.1, 19.7) | 306 (58.0) | 8.8 (-0.4, 17.9) | 868 (51.6) |
| Yes | 25 (67.6) | -10.4 (-50.4, 29.6) | 9 (57.2) | 20.3 (-19.2, 59.7) | 33 (77.5) | 9.9 (-15.9, 35.7) | 67 (70.4) |
| Community-level factors |  |  |  |  |  |  |  |
| Place of residence |  |  |  |  |  |  |  |
| Urban | 23 (56.1) | -1.1 (-26.4, 24.2) | 50 (55.0) | 15.7 (-6.7, 38.1) | 44 (70.7) | 14.6 (-9.8, 39.0) | 118 (60.2) |
| Rural | 277 (49.9) | -2.6 (-12.6, 7.3) | 244 (47.3) | 10.9 (4.4, 21.3) | 296 (58.1) | 8.2 (-1.2, 17.6) | 817 (51.7) |
| Region of residence |  |  |  |  |  |  |  |
| Large central | 278 (50.1) | -1.0 (-11.0, 9.0) | 271 (49.1) | 9.9 (-0.6, 20.4) | 301 (59.0) | 8.9 (-0.7, 18.5) | 850 (52.6) |
| Small peripheral | 16 (47.9) | -17.1 (-33.3, -0.8) | 10 (30.9) | 26.5 (13.8, 39.3) | 25 (57.4) | 9.5 (-6.3, 25.2) | 51 (46.5) |
| Metropolis | 8 (66.9) | -9.7 (-3.2, 13.0) | 13 (57.2) | 22.6 (0.4, 44.9) | 13 (79.8) | 12.9 (-6.0, 31.9) | 34 (66.7) |

**n (%): weighted count and proportion for each outcome variable by study factors**

**Diff-1 indicates percentage point changes from 2005 to 2011; Diff-2 indicates percentage point change from 2011 to 2016; Diff-3 indicates percentage point change from 2005 to 2016**

**** SNNPR = Southern Nations Nationalities and Peoples Region**
