# Supplementary material for: Trends and factors associated with complementary feeding practices in Ethiopia from 2005 to 2016
Source: Matern Child Nutr. 2019 Dec 12;16(2):e12926. doi: 10.1111/mcn.12926 (PMC7083482; doi:10.1111/mcn.12926)
Supplement: Supplementary file 3 — Table S3. Percentage point change in the prevalence of minimum dietary diversity by study factors, 2005–2016 [file MCN-16-e12926-s003.docx]

**Table S3**

Percentage point change in the prevalence of minimum dietary diversity by study factors, 2005–2016

| **Variables** | **2005** | **2005–2011** | **2011** | **2011–2016** | **2016** | **2005–2016** | **2005–2016** |
| --- | --- | --- | --- | --- | --- | --- | --- |
|  | **n (%)** | **Diff -1**  **(95% CI)** | **n (%)** | **Diff-2**  **(95% CI)** | **n (%)** | **Diff-3**  **(95% CI)** | **n (%)** |
| **Socioeconomic factors** |  |  |  |  |  |  |  |
| Maternal education |  |  |  |  |  |  |  |
| No schooling | 96 (4.3) | -2.1 (-3.7, -0.5) | 45 (2.2) | 8.2 (5.4, 11.0) | 189 (10.4) | 6.1 (3.1, 9.1) | 330 (5.5) |
| Primary school | 57 (11.3) | -3.0 (-7.6, 1.5) | 69 (8.3) | 5.8 (1.4, 10.2) | 131 (14.1) | 2.7 (-2.4, 7.9) | 256 (11.3) |
| Secondary and higher | 27 (19.7) | 2.0 (-9.8, 13.8) | 28 (21.7) | 11.7 (-0.9, 24.2) | 82 (33.3) | 13.7 (3.1, 24.3) | 137 (26.8) |
| Maternal occupation |  |  |  |  |  |  |  |
| No occupation | 122 (6.1) | -2.0 (-4.1, 0.1) | 59 (4.1) | 7.9 (4.9, 11.0) | 212 (12.1) | 6.0 (2.8, 9.1) | 393 (7.6) |
| Formal occupation | 29 (12.7) | -5.3 (-11.5, 0.9) | 39 (7.4) | 16.2 (9.6, 22.8) | 112 (23.7) | 11.0 (3.0, 18.9) | 180 (14.7) |
| Informal occupation | 29 (4.5) | -0.1 (-3.1, 2.9) | 43 (4.4) | 5.9 (1.6, 10.2) | 78 (10.3) | 5.8 (12.9, 10.3) | 150 (6.4) |
| Partner education |  |  |  |  |  |  |  |
| No schooling | 65 (4.1) | -1.7 (-3.4, 0.01) | 32 (2.3) | 7.0 (3.9, 10.1) | 106 (8.6) | 5.4 (2.2, 8.5) | 233 (5.3) |
| Primary school | 58 (6.2) | -0.5 (-3.6, 2.5) | 71 (5.7) | 9.1 (4.5, 13.7) | 172 (14.8) | 8.6 (3.7, 13.4) | 301 (9.0) |
| Secondary and higher | 56 (17.6) | -3.8 (-11.5, 3.8) | 35 (13.8) | 10.3 (2.6, 18.1) | 98 (24.1) | 6.5 (-1.5, 14.5) | 189 (19.3) |
| Household wealth status |  |  |  |  |  |  |  |
| Poor | 50 (4.0) | -1.0 (-3.1, 1.1) | 40 (3.0) | 6.1 (3.0, 9.2) | 120 (9.1) | 5.0 (1.8, 8.3) | 211 (5.4) |
| Middle | 28 (4.4) | -1.7 (-5.0, 1.5) | 16 (2.7) | 9.7 (5.3, 14.0) | 81 (12.3) | 7.9 (3.3, 12.5) | 124 (6.6) |
| Rich | 102 (10.3) | -1.9 (-5.2, 1.4) | 85 (8.4) | 11.5 (6.5, 16.6) | 201 (20.0) | 9.6 (4.4, 14.8) | 388 (12.9) |
| **Demographic factors** |  |  |  |  |  |  |  |
| Maternal age |  |  |  |  |  |  |  |
| 15–24 years | 57 (6.9) | -1.2 (-4.4, 2.1) | 49 (5.7) | 6.8 (2.6, 10.9) | 105 (12.5) | 5.6 (1.4, 9.8) | 210 (8.3) |
| 25–34 years | 94 (6.8) | -1.7 (-4.3, 0.9) | 77 (5.1) | 10.0 (6.4, 13.7) | 232 (15.1) | 8.3 (4.4, 12.3) | 403 (9.1) |
| 35–49 years | 29 (4.5) | -1.8 (-4.5, 0.8) | 15 (2.4) | 8.0 (3.7, 12.4) | 65 (10.7) | 6.2 (1.6, 10.8) | 109 (5.9) |
| Listening radio |  |  |  |  |  |  |  |
| No | 73 (4.0) | -1.7 (-3.4, -0.01) | 33 (2.2) | 7.9 (4.4, 10.4) | 220 (10.2) | 6.2 (3.6, 8.8) | 327 (5.9) |
| Yes | 107 (10.5) | -3.1 (-6.4, 0.2) | 108 (7.5) | 14.8 (9.9, 19.8) | 182 (22.3) | 11.8 (6.4, 17.2) | 396 (12.1) |
| Reading newspaper/magazine |  |  |  |  |  |  |  |
| No | 146 (5.5) | -1.6 (-3.3, 0.1) | 104 (3.9) | 7.8 (5.4, 10.2) | 323 (11.6) | 6.2 (3.5, 8.8) | 573 (7.0) |
| Yes | 34 (18.5) | -3.3 (-12.2, 5.6) | 37 (15.3) | 21.7 (10.0, 33.3) | 79 (36.9) | 18.4 (6.3, 30.4) | 150 (23.5) |
| Watching TV |  |  |  |  |  |  |  |
| No | 131 (5.1) | -3.1 (-4.6, -1.5) | 39 (2.0) | 8.8 (6.3, 11.2) | 263 (10.8) | 5.7 (3.1, 8.3) | 433 (6.2) |
| Yes | 49 (18.0) | -7.6 (-14.5, -0.8) | 101 (10.3) | 15.1 (7.8, 22.4) | 139 (25.5) | 7.5 (-1.9, 16.9) | 290 (16.1) |
| Desire for the pregnancy |  |  |  |  |  |  |  |
| Desired the pregnancy | 165 (7.0) | -1.9 (-3.8, 0.01) | 135 (5.1) | 8.0 (5.3, 10.6) | 356 (13.0) | 6.1 (3.2, 8.9) | 656 (8.5) |
| Not desired the pregnancy | 15 (3.0) | -1.0 (-4.1, 2.0) | 6 (2.0) | 15.9 (7.7, 24.1) | 46 (18.0) | 14.9 (6.9, 23.0) | 67 (6.4) |
| **Health service factors** |  |  |  |  |  |  |  |
| Antenatal Visit |  |  |  |  |  |  |  |
| None | 110 (5.4) | -2.9 (-4.9, -0.1) | 42 (2.5) | 9.7 (5.8, 13.6) | 125 (12.2) | 6.8 (2.7, 10.9) | 277 (5.8) |
| 1–3 | 18 (3.9) | 2.4 (-1.1, 5.9) | 46 (6.4) | 4.7 (0.4, 9.0) | 101 (11.1) | 7.1 (3.1, 11.2) | 165 (7.9) |
| 4+ | 52 (14.3) | -4.3 (-10.1, 1.4) | 53 (10.0) | 6.8 (1.8, 11.8) | 173 (16.8) | 2.5 (-3.8, 8.7) | 278 (14.4) |
| Postnatal check-up |  |  |  |  |  |  |  |
| No | 149 (5.5) | -0.8 (-2.5, 0.9) | 134 (4.7) | 8.4 (5.8, 11.1) | 360 (13.1) | 7.6 (4.8, 10.4) | 643 (7.7) |
| Yes | 31 (19.2) | -11.6 (-21.8, -1.3) | 7 (7.6) | 9.7 (-0.6, 19.9) | 42 (17.3) | -1.9 (-12.1, 8.3) | 80 (16.1) |
| Community-level factors |  |  |  |  |  |  |  |
| Place of residence |  |  |  |  |  |  |  |
| Urban | 46 (21.2) | -9.2 (-16.4, -1.9) | 48 (12.0) | 18.1 (8.5, 27.7) | 109 (30.1) | 8.9 (-1.9, 19.7) | 204 (20.7) |
| Rural | 134 (50.1) | -1.4 (-3.1, 0.2) | 92 (3.6) | 7.5 (5.0, 10.1) | 293 (11.2) | 6.1 (3.4, 8.8) | 519 (6.6) |
| Region of residence |  |  |  |  |  |  |  |
| Larger central | 164 (6.2) | -1.6 (-3.5, 0.2) | 124 (4.6) | 8.5 (5.6, 11.3) | 352 (13.0) | 6.8 (3.8, 9.9) | 639 (7.9) |
| Small peripheral | 5 (3.1) | 1.6 (-1.4, 4.6) | 7 (4.7) | 2.3 (-0.9, 5.6) | 13 (7.1) | 3.9 (0.9, 7.0) | 25 (5.1) |
| Metropolis | 11 (18.6) | -6.5 (-15.7, 2.6) | 10 (12.1) | 25.1 (14.9, 35.4) | 37 (37.3) | 18.6 (6.7, 30.6) | 58 (23.9) |

**n (%): weighted count and proportion for each outcome variable by study factors**

**Diff-1 indicates percentage point changes from 2005 to 2011; Diff-2 indicates percentage point change from 2011 to 2016; Diff-3 indicates percentage point change from 2005 to 2016**

**** SNNPR = Southern Nations Nationalities and Peoples Region**
