# Supplementary material for: Trends and factors associated with complementary feeding practices in Ethiopia from 2005 to 2016
Source: Matern Child Nutr. 2019 Dec 12;16(2):e12926. doi: 10.1111/mcn.12926 (PMC7083482; doi:10.1111/mcn.12926)
Supplement: Supplementary file 4 — Table S4. Percentage point change in the prevalence of minimum meal frequency by study factors, 2005–2016 [file MCN-16-e12926-s004.docx]

**Table S3**

Percentage point change in the prevalence of minimum meal frequency by study factors, 2005–2016

| **Variables** | **2005** | **2005–2011** | **2011** | **2011–2016** | **2016** | **2005–2016** | **2005–2016** |
| --- | --- | --- | --- | --- | --- | --- | --- |
|  | **n (%)** | **Diff -1**  **(95% CI)** | **n (%)** | **Diff-2**  **(95% CI)** | **n (%)** | **Diff-3**  **(95% CI)** | **n (%)** |
| **Socioeconomic factors** |  |  |  |  |  |  |  |
| Maternal education |  |  |  |  |  |  |  |
| No schooling | 869 (39.0) | 4.9 (0.3, 9.5) | 875 (43.9) | -1.7 (-7.0, 3.7) | 764 (42.2) | 3.2 (-2.0, 8.5) | 2508 (41.6) |
| Primary school | 235 (47.1) | 9.4 (1.4, 17.5) | 469 (56.6) | -14.6 (-22.0, -7.3) | 391 (41.9) | -5.2 (-13.1, 2.7) | 1094 (48.4) |
| Secondary and higher | 80 (58.3) | 8.0 (-7.8, 23.8) | 84 (66.3) | -6.7 (-20.7, 7.3) | 147 (59.6) | 1.3 (-12.5, 15.1) | 312 (60.9) |
| Maternal occupation |  |  |  |  |  |  |  |
| No occupation | 777 (38.9) | 5.3 (-0.2, 10.8) | 633 (44.2) | -3.7 9-9.7, 2.2) | 709 (40.5) | 1.6 (-3.6, 6.7) | 2119 (40.9) |
| Formal occupation | 131 (56.7) | 2.6 (-8.0 (13.1) | 308 (59.3) | -14.7 (-24.5, -4.8) | 211 (44.6) | -12.1 (-23.9, -0.4) | 650 (53.1) |
| Informal occupation | 274 (43.3) | 5.9 (-1.8, 13.8) | 479 (49.2) | 0.9 (-7.3, 9.1) | 381 (50.1) | 6.9 (-1.9, 15.7) | 1134 (47.9) |
| Partner education |  |  |  |  |  |  |  |
| No schooling | 614 (38.6) | 6.3 (0.9, 11.8) | 635 (44.9) | -3.5 (-9.2, 2.3) | 518 (41.8) | 2.9 (-2.6, 8.4) | 1841 (41.6) |
| Primary school | 393 (41.9) | 8.4 (2.3, 14.5) | 626 (50.4) | -8.1 (-14.2, -2.0) | 491 (42.2) | 0.3 (-6.3, 6.9) | 1509 (45.2) |
| Secondary and higher | 171 (53.1) | 4.9 9-6.3, 16.2) | 147 (58.1) | -2.9 (-13.8, 8.0) | 225 (55.2) | 2.1 (-8.0, 12.1) | 542 (55.3) |
| Household wealth status |  |  |  |  |  |  |  |
| Poor | 468 (37.3) | 7.2 (1.6, 12.9) | 601 (44.5) | -5.8 9-11.6, -0.01) | 512 (38.7) | 1.4 (-4.4, 7.3) | 1581 (40.2) |
| Middle | 250 (40.1) | 11.3 (3.1, 19.4) | 305 (51.3) | -4.0 (-13.7, 5.6) | 310 (47.3) | 7.2 (-1.7, -16.2) | 865 (46.2) |
| Rich | 467 (47.3) | 4.7 (-1.7, 11.1) | 523 (52.0) | -4.5 (-11.4, 2.5) | 479 (47.5) | 0.2 (-6.2, 6.6) | 1468 (44.5) |
| **Demographic factors** |  |  |  |  |  |  |  |
| Maternal age |  |  |  |  |  |  |  |
| 15–24 years | 335 (40.3) | 6.4 (-0.4, 13.2) | 400 (46.7) | -5.9 (-12.8, 1.1) | 343 (40.9) | 0.6 (-6.3, 7.4) | 1078 (42.7) |
| 25–34 years | 560 (40.2) | 9.7 (4.2, 15.1) | 752 (49.9) | -4.5 (-10.1, 1.1) | 697 (45.4) | 5.2 (-0.01, 10.4) | 2009 (45.3) |
| 35–49 years | 290 (45.1) | 2.1 (-5.8, 9.8) | 276 (47.2) | -4.5 (-13.0, 3.9) | 262 (42.7) | -2.5 (-10.4, 5.4) | 828 (45.0) |
| Listening radio |  |  |  |  |  |  |  |
| No | 692 (37.4) | 5.2 (0.2, 10.1) | 639 (42.6) | -0.7 (-5.8, 4.4) | 909 (41.8) | 4.4 (-0.2, 9.0) | 2240 (40.6) |
| Yes | 493 (48.5) | 6.1 (0.4, 11.9) | 789 (54.6) | -6.4 (-13.4, 0.6) | 393 (48.2) | -0.3 (-7.6, 7.0) | 1674 (51.1) |
| Reading newspaper/magazine |  |  |  |  |  |  |  |
| No | 1093 (40.8) | 6.5 (2.5, 10.5) | 1280 (47.3) | -4.3 (-8.7, 0.1) | 1192 (43.0) | 2.2 (-2.1, 6.5) | 3564 (43.7) |
| Yes | 89 (49.0) | 12.7 (-0.8, 26.3) | 149 (61.7) | -10.7 (-23.0, 1.6) | 110 (51.1) | 2.0 (-10.7, 14.8) | 348 (54.5) |
| Watching TV |  |  |  |  |  |  |  |
| No | 1048 (40.5) | 4.5 (0.1, 9.0) | 885 (45.0) | -3.6 (-8.4, 11.8) | 1010 (41.4) | 0.9 (-3.5, 5.3) | 2942 (42.1) |
| Yes | 134 (49.2) | 5.9 (-3.6, 15.5) | 541 (55.1) | -1.8 (-9.7, 6.0) | 291 (53.3) | 4.1 (-6.3, 14.6) | 967 (53.7) |
| Desire for the pregnancy |  |  |  |  |  |  |  |
| Desired the pregnancy | 959 (40.7) | 8.8 (4.8, 12.9) | 1316 (49.5) | -6.1 (-10.5, -1.8) | 1186 (43.4) | 2.7 (-1.6, 7.0) | 3461 (44.7) |
| Not desired the pregnancy | 226 (44.6) | -6.2 (-17.4, 5.0) | 113 (38.4) | 6.9 (-6.6, 20.5) | 116 (45.3) | 7.6 (-10.6, 12.1) | 454 (43.1) |
| **Health service factors** |  |  |  |  |  |  |  |
| Antenatal Visit |  |  |  |  |  |  |  |
| None | 809 (39.9) | 3.7 9-1.1, 8.6) | 736 (43.6) | -3.5 (-9.9, 3.0) | 410 (40.1) | -0.3 (-6.0, 6.6) | 1956 (41.2) |
| 1–3 | 181 (39.7) | 12.5 (4.3, 20.8) | 376 (52.2) | -8.1 (-15.3, -0.8) | 403 (44.1) | 4.5 (-3.4, 12.3) | 961 (45.9) |
| 4+ | 187 (51.9) | 7.2 (-2.0, 16.6) | 315 (59.1) | -12.9 (-20.3, -5.6) | 477 (46.2) | -5.7 (-14.1, 2.8) | 979 (50.8) |
| Postnatal check-up |  |  |  |  |  |  |  |
| No | 1095 (40.5) | 7.7 (3.7, 11.7) | 1378 (48.2) | -4.9 (-9.4, -0.5) | 1189 (43.3) | 2.8 (-1.5, 7.1) | 3662 (44.1) |
| Yes | 89 (54.9) | -0.3 (-16.9, 16.4) | 51 (54.7) | -7.9 (-24.4, 8.6) | 113 (46.8) | -8.2 (-20.9, 4.6) | 252 (50.9) |
| Community-level factors |  |  |  |  |  |  |  |
| Place of residence |  |  |  |  |  |  |  |
| Urban | 115 (52.6) | -0.6 (-12.4, 11.3) | 210 (52.0) | 3.9 (-7.2, 15.0) | 203 (56.0) | 3.4 (-7.0, 13.8) | 529 (53.6) |
| Rural | 1069 (40.4) | 7.5 (3.3, 11.6) | 1218 (47.9) | -6.0 (-10.5, -1.5) | 1098 (41.9) | 1.4 (-2.9, 5.8) | 3386 (43.3) |
| Region of residence |  |  |  |  |  |  |  |
| Large central | 1101 (41.7) | 7.5 (3.4, 11.7) | 1340 (49.2) | -6.0 (-10.6, -1.4) | 1165 (43.2) | 1.5 (-2.9, 6.0) | 3606 (44.7) |
| Small peripheral | 46 (28.1) | 1.1 (-6.3, 8.6) | 42 (29.3) | 11.2 (4.1, 18.4) | 77 (40.5) | 12.4 (4.9, 19.8) | 165 (33.2) |
| Metropolis | 37 (61.8) | -6.5 (-20.4, 7.4) | 47 (55.3) | 4.1 (-10.7, 18.8) | 59 (59.3) | 2.4 (-14.2, 9.3) | 143 (58.5) |

**n (%): weighted count and proportion for each outcome variable by study factors**

**Diff-1 indicates percentage point changes from 2005 to 2011; Diff-2 indicates percentage point change from 2011 to 2016; Diff-3 indicates percentage point change from 2005 to 2016**

**** SNNPR = Southern Nations Nationalities and Peoples Region**
