# Supplementary material for: Trends and factors associated with complementary feeding practices in Ethiopia from 2005 to 2016
Source: Matern Child Nutr. 2019 Dec 12;16(2):e12926. doi: 10.1111/mcn.12926 (PMC7083482; doi:10.1111/mcn.12926)
Supplement: Supplementary file 5 — Table S5. Percentage point change in the prevalence of minimum acceptable diet by study factors, 2005–2016 [file MCN-16-e12926-s005.docx]

**Table S5**

Percentage point change in the prevalence of minimum acceptable diet by study factors, 2005–2016

| **Variables** | **2005** | **2005–2011** | **2011** | **2011–2016** | **2016** | **2005–2016** | **2005–2016** |
| --- | --- | --- | --- | --- | --- | --- | --- |
|  | **n (%)** | **Diff -1**  **(95% CI)** | **n (%)** | **Diff-2**  **(95% CI)** | **n (%)** | **Diff-3**  **(95% CI)** | **n (%)** |
| **Socioeconomic factors** |  |  |  |  |  |  |  |
| Maternal education |  |  |  |  |  |  |  |
| No schooling | 60 (2.7) | -0.7 (-2.0, 0.7) | 40 (2.0) | 3.2 (1.2, 5.3) | 94 (5.2) | 2.6 (4.4, 4.7) | 194 (3.2) |
| Primary school | 42 (8.4) | -1.4 (-5.5, 2.6) | 58 (7.0) | -0.1 (-3.5, 3.2) | 64 (6.8) | -1.6 (-5.5, 2.3) | 164 (7.3) |
| Secondary and higher | 15 (11.3) | 6.3 (-4.9, 17.4) | 22 (17.5) | 4.0 (-8.5, 16.5) | 53 (21.6) | 10.3 (-0.5, 21.1) | 91 (17.8) |
| Maternal occupation |  |  |  |  |  |  |  |
| No occupation | 74 (3.7) | -0.2 (-1.9, 1.6) | 51 (3.5) | 2.5 (0.2, 4.8) | 106 (6.0) | 2.3 (1.9, 4.5) | 230 (4.4) |
| Formal occupation | 20 (8.8) | -2.8 (-8.2, 2.7) | 31 (6.0) | 8.5 (2.2, 14.8) | 69 (14.5) | 5.7 9-1.6, 13.1) | 120 (9.8) |
| Informal occupation | 23 (3.7) | 0.2 (-2.3, 2.9) | 38 (3.9) | 1.0 (-2.0, 4.0) | 37 (4.9) | 1.2 (-1.9, 4.4) | 99 (4.2) |
| Partner education |  |  |  |  |  |  |  |
| No schooling | 41 (2.6) | -0.3 (-1.9, 1.2) | 29 (2.1) | 2.0 (-0.3, 4.3) | 50 (4.0) | 1.7 (-0.6, 4.0) | 133 (3.0) |
| Primary school | 41 (4.3) | 0.6 (-1.9, 3.1) | 62 (5.0) | 2.1 (-1.0, 5.1) | 82 (7.0) | 2.7 (-0.4, 5.8) | 184 (5.5) |
| Secondary and higher | 35 (11.0) | -0.7 (-7.7, 6.2) | 26 (10.3) | 7.0 (-0.6, 14.6) | 71 (17.3) | 6.3 (-1.2, 13.8) | 132 (13.4) |
| Household wealth status |  |  |  |  |  |  |  |
| Poor | 35 (2.8) | -0.3 (-2.1, 1.5) | 33 (2.4) | 2.2 (0.01, 4.4) | 62 (4.7) | 1.9 (-0.4, 4.2) | 129 (3.3) |
| Middle | 17 (2.7) | -0.1 (-3.1, 2.9) | 16 (2.7) | 5.5 (1.9, 9.2) | 54 (8.2) | 5.5 (1.7, 9.3) | 87 (4.6) |
| Rich | 66 (6.7) | 0.4 (-2.4, 3.3) | 71 (7.1) | 2.5 (-1.4, 6.3) | 96 (9.6) | 2.9 (-0.1, 6.6) | 233 (7.8) |
| **Demographic factors** |  |  |  |  |  |  |  |
| Maternal age |  |  |  |  |  |  |  |
| 15–24 years | 39 (4.7) | -.7 (-2.3, 3.7) | 46 (5.4) | 1.5 (-2.0, 5.0) | 57 (6.9) | 2.2 (-1.2, 5.6) | 142 (5.6) |
| 25–34 years | 60 (4.3) | -0.3 (-2.4, 1.9) | 61 (4.1) | 4.2 (1.4, 7.1) | 127 (8.3) | 4.0 (1.1, 6.9) | 248 (5.6) |
| 35–49 years | 18 (2.9) | -0.6 (-3.0, 1.7) | 13 (2.2) | 2.2 (-0.8. 5.1) | 27 (4.4) | 1.5 (-1.5, 4.5) | 58 (3.2) |
| Listening radio |  |  |  |  |  |  |  |
| No | 47 (2.5) | -0.6 (-2.1, 8.3) | 28 (1.9) | 3.8 (1.9, 5.7) | 124 (5.7) | 3.2 (1.3, 5.1) | 199 (3.6) |
| Yes | 71 (7.0) | -0.6 (-3.4, 2.1) | 92 (6.3) | 4.4 (0.5, 8.4) | 89 (10.8) | 3.8 (-0.4, 8.0) | 250 (7.6) |
| Reading newspaper/magazine |  |  |  |  |  |  |  |
| No | 96 (3.6) | -0.2 (-1.6, 1.3) | 92 (3.4) | 2.6 (7.7, 4.5) | 167 (6.0) | 2.5 (0.6, 4.4) | 355 (4.4) |
| Yes | 21 (11.8) | -0.3 (-7.8, 7.3) | 28 (11.5) | 9.1 (-1.8, 20.0) | 44 (20.6) | 8.8 (-2.0, 19.7) | 93 (14.7) |
| Watching TV |  |  |  |  |  |  |  |
| No | 93 (3.6) | -1.8 (-3.1, -0.4) | 36 (1.8) | 3.7 (2.0, 5.4) | 135 (5.5) | 1.9 (0.2, 3.7) | 263 (3.8) |
| Yes | 24 (9.0) | -0.4 (-5.9, 5.2) | 84 (8.6) | 5.4 (-0.3, 11.1) | 76 (14.0) | 5.0 (-2.1, 12.2) | 185 (10.3) |
| Desire for the pregnancy |  |  |  |  |  |  |  |
| Desired the pregnancy | 104 (4.4) | -0.1 (-1.7, 1.4) | 114 (4.3) | 2.5 (0.6, 4.5) | 187 (6.8) | 2.4 (0.4, 4.4) | 405 (5.2) |
| Not desired the pregnancy | 13 (2.6) | -0.6 (-3.6, 2.5) | 6 (2.0) | 7.8 91.6, 14.0) | 25 (9.8) | 7.2 (1.3, 13.2) | 44 (4.2) |
| **Health service factors** |  |  |  |  |  |  |  |
| Antenatal Visit |  |  |  |  |  |  |  |
| None | 809 (39.9) | --1.2 (-2.8, 0.4) | 37 (2.2) | 4.6 (1.7, 7.6) | 70 (6.8) | 3.5 (0.5, 6.4) | 174 (3.7) |
| 1–3 | 181 (39.7) | 2.7 (-0.6, 6.1) | 41 (5.8) | -0.4 (-3.9, 3.0) | 49 (5.3) | 2.3 (-0.7, 5.3) | 104 (5.0) |
| 4+ | 187 (51.9) | -1.7 (-6.8, 3.4) | 42 (7.9) | 1.1 (-3.1, 5.4) | 93 (9.0) | -0.6 (-5.8, 4.6) | 170 (8.8) |
| Postnatal check-up |  |  |  |  |  |  |  |
| No | 98 (3.6) | 3.4 (-10.5, 17.3) | 114 (4.0) | 2.9 (0.9, 4.9) | 189 (6.9) | 3.3 (1.3, 5.2) | 401 (4.8) |
| Yes | 19 (11.5) | -4.7 (-13.9, 4.3) | 6 (6.8) | 2.4 (-6.9, 11.7) | 22 (9.2) | -2.4 (-10.4, 5.7) | 47 (9.5) |
| Community-level factors |  |  |  |  |  |  |  |
| Place of residence |  |  |  |  |  |  |  |
| Urban | 24 (11.0) | -1.7 (-8.0, 4.5) | 37 (9.2) | 9.0 (1.7, 16.3) | 66 (18.2) | 7.2 (-1.0, 15.5) | 128 (12.9) |
| Rural | 93 (3.5) | -0.3 (-1.7, 11.3) | 83 (3.3) | 2.3 (4.7, 4.1) | 145 (5.5) | 2.0 (0.2, 3.9) | 321 (4.1) |
| Region of residence |  |  |  |  |  |  |  |
| Large central | 109 (4.1) | -0.1 (-1.6, 1.5) | 111 (4.1) | 2.6 (0.6, 4.7) | 181 (6.7) | 2.6 (0.4, 4.7) | 400 (5.0) |
| Small peripheral | 2 (1.1) | 1.9 (-0.1, 3.9) | 4 (3.0) | 1.6 (-0.9, 4.2) | 9 (4.7) | 3.6 (1.7, 5.4) | 15 (3.0) |
| Metropolis | 6 (10.7) | -4.6 (-11.8, 2.7) | 5 (6.1) | 16.0 (7.5, 24.6) | 22 (22.1) | 11.5 (1.1, 21.8) | 33 (13.7) |

**n (%): weighted count and proportion for each outcome variable by study factors**

**Diff-1 indicates percentage point changes from 2005 to 2011; Diff-2 indicates percentage point change from 2011 to 2016; Diff-3 indicates percentage point change from 2005 to 2016**

**** SNNPR = Southern Nations Nationalities and Peoples Region**
